# Differential requirements for MDM2 E3 activity during embryogenesis and in adult mice

**DOI:** 10.1101/gad.341875.120

**Published:** 2021-01-01

**Authors:** Timothy J. Humpton, Koji Nomura, Julia Weber, Helge M. Magnussen, Andreas K. Hock, Colin Nixon, Sandeep Dhayade, David Stevenson, Danny T. Huang, Douglas Strathdee, Karen Blyth, Karen H. Vousden

**Affiliations:** 1 The Francis Crick Institute, London NW1 1AT, United Kingdom;; 2 Cancer Research UK Beatson Institute, Glasgow G61 1BD, United Kingdom;; 3 Institute of Cancer Sciences, University of Glasgow, Glasgow G61 1QH, United Kingdom

**Keywords:** MDM2, p53, E3 activity, RING mutant

## Abstract

The p53 tumor suppressor protein is a potent activator of proliferative arrest and cell death. In normal cells, this pathway is restrained by p53 protein degradation mediated by the E3-ubiquitin ligase activity of MDM2. Oncogenic stress releases p53 from MDM2 control, so activating the p53 response. However, many tumors that retain wild-type p53 inappropriately maintain the MDM2-p53 regulatory loop in order to continuously suppress p53 activity. We have shown previously that single point mutations in the human MDM2 RING finger domain prevent the interaction of MDM2 with the E2/ubiquitin complex, resulting in the loss of MDM2's E3 activity without preventing p53 binding. Here, we show that an analogous mouse MDM2 mutant (MDM2 I438K) restrains p53 sufficiently for normal growth but exhibits an enhanced stress response in vitro. In vivo*,* constitutive expression of MDM2 I438K leads to embryonic lethality that is rescued by p53 deletion, suggesting MDM2 I438K is not able to adequately control p53 function through development. However, the switch to I438K expression is tolerated in adult mice, sparing normal cells but allowing for an enhanced p53 response to DNA damage. Viewed as a proof of principle model for therapeutic development, our findings support an approach that would inhibit MDM2 E3 activity without preventing MDM2/p53 binding as a promising avenue for development of compounds to activate p53 in tumors with reduced on-target toxicities.

## Abstract (precis)

In this study, Humpton et al. present a mouse MDM2 mutant (MDM2 I438K) analogous to human cancer cell mutations that restrains p53 sufficiently for normal growth, but exhibits an enhanced stress response in vitro. Their results are a proof of principle model for therapeutic development, and promote an approach that would inhibit MDM2 E3 activity without preventing MDM2/p53 binding.

The p53 transcription factor is a critical regulator of the cellular response to an extensive range of genotoxic, metabolic, and environmental stressors (Levine 2020). The predominant activity of p53 is as a tumor suppressor, and mutations in p53 or defects in the pathways leading to the activation of p53 are the most frequent alterations in cancer, contributing to the development of most tumors (Kastenhuber and Lowe 2017).

Although p53 is ubiquitously expressed, it is restrained from undue action by rapid turnover of the p53 protein. Two related RING finger E3-ligase proteins, MDM2 and MDMX (MDM4 in mice), act as the main negative regulators of p53 stability (Karni-Schmidt et al. 2016; Moyer et al. 2017). Both MDM2 and MDM4 are required to limit p53 activity (Huang et al. 2011; Pant et al. 2011) and in mice the deletion of either results in embryonic lethality that is rescued by concomitant deletion of *Trp53* (Jones et al. 1995; Montes de Oca Luna et al. 1995; Parant et al. 2001). While deletion of *Mdm2* is similarly fatal in adult mice, again in a p53-dependent manner (Ringshausen et al. 2006; Zhang et al. 2014), transient loss of *Mdm4* is tolerated in adult mice expressing wild-type p53, despite the resulting induction of a p53 response (Garcia et al. 2011).

MDM2 and MDMX regulate p53 through a number of mechanisms (Shadfan et al. 2012; Wang and Jiang 2012), including an ability to direct p53 degradation (Haupt et al. 1997; Honda et al. 1997; Kubbutat et al. 1997). In otherwise unstressed cells, MDM2 is predominantly nuclear and exists as either a homodimer or heterodimer, with a second MDM2 molecule, or with MDMX. In either case, it is the MDM2 RING finger domain that is essential for the E3 ubiquitin ligase (E3) activity of either dimer to ubiquitinate p53 and facilitate its proteasome-mediated degradation, as MDMX does not possess intrinsic E3 activity (Linares et al. 2003; Iyappan et al. 2010). Independent of degradation, the binding of MDM2 to the N-terminal transactivation domain of p53 can also directly inhibit p53 activity by restricting the interactions between p53 and other transcriptional machinery (Momand et al. 1992; Oliner et al. 1993; Shi and Gu 2012). Additionally, MDM2 E3 activity can indirectly limit p53 transcriptional activity by ubiquitinating histones within promoters, thereby blocking access for p53 and the transcriptional machinery (Minsky and Oren 2004). The regulation of p53 by MDM2 can therefore be divided into E3-dependent and E3-independent actions that act coordinately to limit the level and subsequent activity of p53 in unstressed cells.

Signals that activate p53 lead to an array of responses that function to block the ability of MDM2 to degrade p53 (Karni-Schmidt et al. 2016). The subsequent accumulation of active p53 allows the transcription of p53 target genes to orchestrate the cellular stress response. Importantly, transcription of *MDM2* itself is also activated by p53, facilitating a self-limiting p53-MDM2 feedback loop that brings p53 signaling back under control when the initiating stress has resolved. In the 30%–50% of human cancers that retain wild-type p53, this loop is compromised, leading to a failure to properly restrain MDM2 and causing continuous limitation of p53 activity irrespective of activating signals (Wasylishen and Lozano 2016). Uncovering mechanisms that would lead to the reactivation of the latent p53 in these tumors is an area of therapeutic interest and has resulted in the development of many compounds that block the MDM2-p53 interaction (Cheok and Lane 2017). However, these compounds have been of limited efficacy in clinical trials due to dose-limiting on-target toxicities arising from p53-mediated neutropenia and thrombocytopenia (Andreeff et al. 2016; Kastenhuber and Lowe 2017).

Previous studies have identified the RING domain and C terminus of MDM2 as critical for E3 activity. The human MDM2 RING domain amino acid C464 is required for MDM2 dimerization and for maintaining overall RING domain structure (Honda and Yasuda 2000), while the C-terminal tail of either MDM2 (in a homodimer) or MDMX (in a heterodimer) are required to support ubiquitin binding (Nomura et al. 2017). Mutation of residue C464 (C462 in mouse MDM2) disrupts both E3-dependent and E3-independent activities of MDM2 and leads to embryonic lethality when expressed in mice (Itahana et al. 2007). Tail region mutations of MDM2 also inhibit the E3 activity of a homodimer, but are active as a heterodimer with wild-type MDMX (Poyurovsky et al. 2007; Singh et al. 2007; Uldrijan et al. 2007). In vivo*,* expression of a C-terminal tail mutant of MDM2 (Y487A) that is defective for E3 activity as a homodimer but can be rescued by wild-type MDMX did not impede embryogenesis, reflecting a retention of the ability to control p53 during development (Tollini et al. 2014).

To examine the importance of complete loss of MDM2 E3 activity, we previously demonstrated that single point mutations in the MDM2 RING finger domain at amino acid I440 or R479 prevented the interaction of MDM2 with the E2/ubiquitin complex, resulting in the loss of MDM2 E3 activity without otherwise disrupting the structure of the MDM2 RING finger domain (Nomura et al. 2017). Although these mutants retained the ability to dimerize, they could not be rescued by interaction with MDMX. Despite compromised E3 function, the MDM2 I440K mutant was able to limit p53 activity in vitro, allowing cells expressing this mutant MDM2 to proliferate. However, cells expressing MDM2 I440K showed clear accumulation of p53 and an enhanced cellular stress response, allowing for more rapid activation of p53 signaling in response to genotoxic stress. These results indicated that under normal tissue culture conditions, the binding of MDM2 to p53 is sufficient to control p53 function, but that this regulation may be more sensitive to activation in response to stress. To explore the in vivo consequences of selective loss of MDM2's E3 activity, we generated a knock-in mouse model of the murine MDM2 mutant analogous to the MDM2 I440K human mutant (MDM2 I438K) and analyzed the effects of this mutant on the p53-MDM2 regulatory loop during murine development, under normal physiological conditions, and in response to DNA damage.

## Results

### Loss of MDM2 E3 activity is not detrimental to primary murine cell proliferation

To understand the in vivo consequences of loss of E3 activity in MDM2 in mice, we first generated mutations in mouse *Mdm2* at residues analogous to human MDM2 C464, I440, and R479 (C462A, I438K, I438E, and R477P), which were previously shown to abrogate E3 function. Transfection of these mutants with p53 into mouse embryo fibroblasts (MEFs) confirmed a similar defect in the ability to degrade p53 compared with wild-type MDM2 (Supplemental Fig. S1A,B). To take this analysis further, we focused on the I438K mutant. Surface plasmon resonance (SPR) analysis of MDM2 WT and MDM2 I438K GST-tagged mouse MDM2 RING domains showed that the I438K mutant lost the ability to bind UbcH5B-Ub, while an in vitro autoubiquitination assay confirmed that the I438K mutant was defective for E3 activity (Supplemental Fig. S1C,D). As expected, MDM2 I438K similarly failed to enhance ubiquitination of p53 compared with the wild-type MDM2 protein in vitro (Supplemental Fig. S1E). However, the I438K mutant retained the ability to interact with both MDM4 (Supplemental Fig. S1F) and p53 (Supplemental Fig. S1G) similarly to wild-type MDM2. Taken together, the data confirm similar profiles of activity for mouse MDM2 I438K as previously described for human MDM2 I440K.

**Figure 1.**
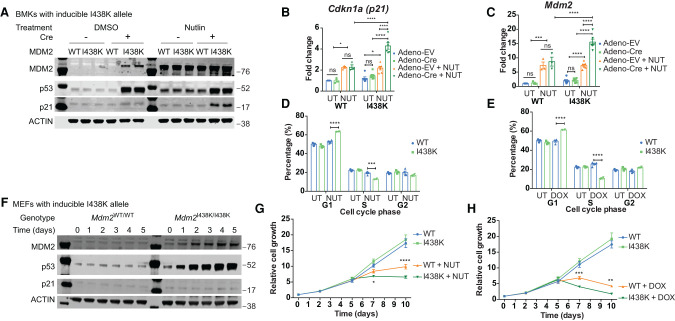
Mouse *Mdm2*^I438K^ restrains basal p53 but allows for an enhanced p53 response in vitro. (*A*) Western blot analysis after 3-h treatment with control (DMSO) or Nutlin (4 μM) of *Mdm2*^I438K/I438K^ (I438K) and *Mdm2*^WT/WT^ (WT) BMK cells first treated for 24 h with Adeno-empty (EV) or Adeno-Cre and left for 5 d prior to experiment. All proteins detected on one blot with ACTIN used as loading control. Protein MW ladder (in kilodaltons) as depicted. Representative blot from three independent experiments. (*B*,*C*) RT-qPCR analysis of expression of p53 target genes *Cdkn1a* (*p21*) (*B*) and *Mdm2* (*C*) after 1-h treatment with DMSO (UT) or 4 μM Nutlin (NUT) in BMK cells as in *A*. *N* = 3/6 WT/I438K-independent samples. Data presented as mean ± SEM and analyzed using two-way ANOVA with Holm-Sidak's multiple comparisons test and multiplicity-adjusted *P*-values. (*D*,*E*) Cell cycle distribution of *Mdm2*^I438K/I438K^ (I438K) and *Mdm2*^WT/WT^ (WT) BMK cells first treated for 24 h with Adeno-Cre, left for 5 d, and then with DMSO control (UT) or with 4 μM Nutlin (NUT) (*D*) or 1 μM doxorubicin (DOX) (*E*) for 8 h prior to analysis. *N* = 3 UT, *N* = 4 NUT/DOX WT, *N* = 2 NUT/DOX I438K-independent samples. Same UT data shown in both *D* and *E*. Data presented as mean ± SEM and analyzed using two-way ANOVA with Holm-Sidak's multiple comparisons test and multiplicity-adjusted *P*-values. (*F*) Western blot analysis in *Rosa*Cre^ER^; *Mdm2*^I438K/I438K^ (I438K) and *Rosa*Cre^ER^; *Mdm2*^WT/WT^ (WT) MEFs for 5 d after treatment with 1 μM 4-OHT at day 0. All proteins detected on one blot with ACTIN used as loading control. Protein MW ladder (in kilodaltons) as depicted. Representative blot from three independent experiments. (*G*,*H*) Relative cell growth of MEFs from *F* treated with DMSO (*G*) or H_2_O (*H*) vehicle control or with 4 μM Nutlin (NUT) (*G*) or 1 μM doxorubicin (DOX) (*H*). *N* = 3 independent replicates/condition. Data presented as mean ± SEM and analyzed using two-way ANOVA with Holm-Sidak's multiple comparisons test and multiplicity-adjusted *P*-values.

To assess the consequences of selective loss of MDM2's ability to interact with E2/ubiquitin on the p53 pathway in vivo, we generated a conditional knock-in mouse expressing the I438K mutant using the approach illustrated in Supplemental Figure S1H. In brief, the targeted allele (tm1 allele) introduced a second copy of the last two exons of WT *Mdm2* (Exons 11 and 12) containing the I438K mutation (Supplemental Fig. S1H). Subsequent excision of the Neo cassette was achieved using FLP-FRT recombination (tm1.1 allele). Cre recombinase-mediated excision of the wild-type exons 11 and 12 leads to the expression of endogenous *Mdm2* carrying the I438K mutation (tm1.2 allele). Baby mouse kidney cells (BMKs) isolated from mice carrying the tm1 allele were used for initial validation of the activity of the I438K allele in vitro. As seen in the transient MEF system, adenoviral Cre-induced expression of MDM2 I438K resulted in robust accumulation of p53, reflecting the loss of MDM2's ability to target p53 for degradation (Fig. 1A). In this model, a more modest accumulation of the MDM2 I438K protein was also detected, consistent with the previously described auto-ubiquitination activity of MDM2 (Fang et al. 2000; Honda and Yasuda 2000). Importantly, induction of MDM2 I438K did not lead to an increase in the expression of *Cdkn1a* (*p21)*, a transcriptional target of p53, at either protein (p21) or mRNA levels (Fig. 1A/B; Supplemental Fig. S1I). Consistent with previous work, these results show that the elevated level of p53 seen in MDM2 I438K-expressing BMK cells was not transcriptionally active.

To interrogate the p53 response in cells expressing MDM2 I438K, we treated BMK cells with Nutlin-3a (Nutlin), a small molecule that inhibits the MDM2-p53 interaction to activate p53 (Vassilev et al. 2004). While treatment of wild-type MDM2-expressing cells with Nutlin for 3 h led to the stabilization of p53, as expected, treatment for this short time did not translate into increased p21 protein levels. In contrast, at 3 h after Nutlin treatment, MDM2 I438K-expressing BMK cells already showed a significant increase in p21 expression (Fig. 1A), consistent with the enhanced sensitivity of these cells to p53 activating signals previously reported in the human system (Nomura et al. 2017). Direct analysis of mRNA confirmed the ability of MDM2 I438K to restrain the ability of p53 to activate expression of the p53 target genes *Cdkn1a (p21)* and *Mdm2* in otherwise unstressed BMK cells (Fig. 1B,C). However, these cells showed a more robust activation of a p53 transcriptional response within 1 h of Nutlin treatment compared with cells expressing wild-type p53 (Fig. 1B,C). A similarly rapid response to the DNA-damaging agent doxorubicin was seen in the activation of *Cdkn1a (p21)* and *Bax* expression (Supplemental Fig. S1I,J). Extending the duration of treatment confirmed that the wild-type p53-expressing cells also responded to these doses of Nutlin and doxorubicin by activating expression of *p21* and *Mdm2* (Supplemental Fig. S1K–N). Compromised induction of *Cdkn1a (p21)* and *Mdm2* in p53-null cells demonstrated the p53 dependence of these responses (Supplemental Fig. S1K–N). Consistent with the lack of transcriptional response, the expression of MDM2 I438K did not impact cell cycle distribution of BMK cells under normal growth conditions (Fig. 1D,E). However, in response to p53 activation by Nutlin or doxorubicin, the rapidly increased p21 expression evident in MDM2 I438K-expressing cells was accompanied by a clear increase of cells in G1 and a reduction in cells in S-phase of the cell cycle. This response was seen within 8 h of treatment, at a time point where no changes in the cell cycle were yet seen in MDM2 WT cells (Fig. 1D,E).

Finally, we derived MEFs from mice carrying both the inducible *Mdm2*^I438K^ tm1.1 allele (*Mdm2*^I438K/I438K^) and a tamoxifen-inducible *Rosa26*-CreERT2 (*Rosa*-Cre^ER^) allele (Hameyer et al. 2007) and assessed their response to p53 induction. As in BMK cells, the induction of mutant MDM2 in response to Cre recombinase activation by 4-hydroxytamoxifen (4-OHT) resulted in an accumulation of p53 protein (Fig. 1F). Under unstressed conditions, MEFs expressing MDM2 I438K grew similarly to MDM2 wild-type cells, yet they showed an accelerated proliferative arrest in response to Nutlin or doxorubicin treatment (Fig. 1G,H). Combined with our BMK data, these findings confirm that the mouse MDM2 I438K mutant fails to target p53 for degradation, but is able to restrain transcription from elevated p53 in unstressed situations. However, cells expressing this mutant show a rapid and robust p53-mediated response to stress in vitro.

### Loss of MDM2 E3 activity leads to embryonic lethality but is tolerated in adult mice

**Figure 2.**
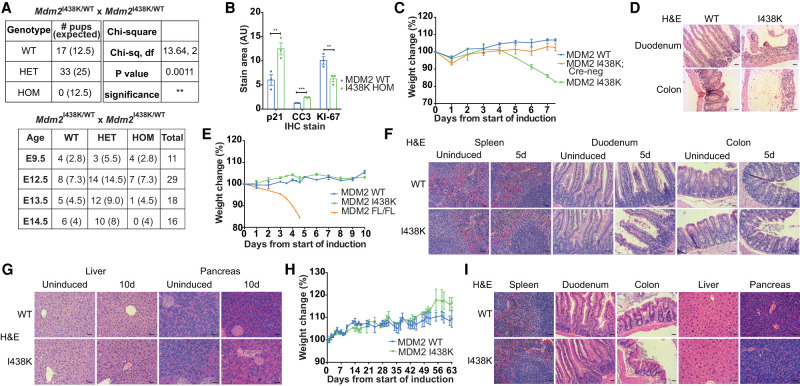
Inducible switch to *Mdm2*^*I438K*^ expression is viable in adult mice. (*A*) Genotypes of 50 pups born to *Mdm2*^I438K/WT^ X *Mdm2*^I438K/WT^ matings (offspring from five mating pairs) and the gestational age and genotypes of embryos obtained from 10 timed matings of *Mdm2*^I438K/WT^ X *Mdm2*^I438K/WT^ mice. *Mdm2*^I438K/I438K^ mice denoted as “HOM.” Litter data analyzed using a χ^2^ test as shown. Brackets denote expected frequencies. (*B*) Quantification of p21, cleaved caspase 3 (CC3), and KI-67 staining in E12.5 *Mdm2^WT/WT^* (MDM2 WT) and *MDM2^I438K/I438K^* (I438K HOM) embryos. *N* = 3 embryos/group derived from three timed matings. Data analyzed using multiple *t*-tests with Holm-Sidak's method to correct for multiple comparisons. (*C*) Weights from *Rosa*Cre^ER^; *Mdm2*^*I*^^438K/I438K^ (I438K), *Mdm2*^I438K/I438K^ (I438K no Cre), and *Mdm2*^WT/WT^ (WT) (two Cre-positive, one negative) mice during IP tamoxifen induction presented as percent change from preinduction baseline measurement. *N* = 3 mice per group. Data are presented as mean ± SEM. (*D*) Representative H&E staining of duodenum and colon of *Rosa*Cre^ER^; *Mdm2*^I438K/I438K^ (I438K) and *Rosa*Cre^ER^; *Mdm2*^WT/WT^ (WT) mice from *C*. Scale bar, 20 μm. (*E*) Weights from *Rosa*Cre^ER^; Mdm2^I438K/I438K^ (I438K), *Rosa*Cre^ER^; *Mdm2*^WT/WT^ (WT), and *Rosa*Cre^ER^; *Mdm2*^FL/FL^ (FL/FL) mice during SC tamoxifen induction protocol presented as percent change from preinduction baseline measurement. *N* = 4 WT/I438K and *N* = 1 FL/FL. Data are presented as mean ± SEM. (*F*) Representative H&E staining of proliferative tissues in uninduced mice and at 5 d after initial dose of SC tamoxifen treatment in *Rosa*Cre^ER^; *Mdm2*^I438K/I438K^ (I438K) and *Rosa*Cre^ER^; *Mdm2*^WT/WT^ (WT) mice. Scale bar, 20 μm. *N* = 7 WT and *N* = 6 I438K uninduced mice and *N* = 4 induced mice per group at 5 d after induction. (*G*) Representative H&E staining of nonproliferative tissues as in *F* at 10 d after initial dose of SC tamoxifen induction. Scale bar, 20 μm. *N* = 7 WT and *N* = 6 I438K uninduced mice and *N* = 6 WT and *N* = 5 I438K induced mice at 10 d per group. (*H*) Long-term weight measurements from male *Rosa*Cre^ER^; *Mdm2*^I438K/I438K^ (I438K) and *Rosa*Cre^ER^; *Mdm2*^WT/WT^ (WT) mice after SC tamoxifen induction protocol presented as percent change from preinduction baseline measurement. *N* = 4 WT and *N* = 2 I438K (all males). Data presented as mean ± range. (*I*) Representative H&E staining from indicated tissues of aged *Rosa*Cre^ER^; *Mdm2*^I438K/I438K^ (I438K) and *Rosa*Cre^ER^; *Mdm2*^WT/WT^ (WT) mice from *H*. *N* = 4 WT and *N* = 2 I438K mice. Scale bar, 20 μm.

Having established that the expression of MDM2 I438K was not detrimental to the proliferation of cells under unstressed conditions in vitro, we looked at the consequences of expression of this MDM2 mutant during mouse development. In initial experiments, we crossed mice carrying the inducible *Mdm2*^I438K^ mutant allele (tm1) with mice carrying a ubiquitously expressed Deleter-Cre construct (Schwenk et al. 1995), inducing constitutive heterozygous expression of *Mdm2*^I438K^ (*Mdm2*^I438K/WT^) in ensuing offspring. Resulting *Mdm2*^I438K/WT^ mice were intercrossed to generate *Mdm2*^I438K/I438K^ homozygous mice. None of the pups born from this cross were homozygous for the mutant allele, suggesting that expression of MDM2 I438K is embryonically lethal (Fig. 2A). A closer examination of embryos from this mating strategy showed that *Mdm2*^I438K/I438K^ mice survived until E12.5 but were subsequently undetectable by E14.5 (Fig. 2A). Histology of embryos wild-type or homozygous for *Mdm2*^*I438K*^ at E12.5 did not reveal any clear morphological differences (Supplemental Fig. S2A). However, IHC showed a clear increase in expression of the p53 target gene *Cdkn1a* (*p21)*, which was accompanied by increased cleaved caspase 3 (CC3) staining, indicating enhanced apoptosis, and decreased expression of the proliferation marker KI-67 in the I438K homozygous embryos (Fig. 2B; Supplemental Fig. S2A). For comparison, MDM2-null embryos die through apoptosis around E3.5 (Jones et al. 1995; Montes de Oca Luna et al. 1995; Chavez-Reyes et al. 2003), while mice carrying a RING domain-disrupting MDM2 mutant (MDM2 C462A) die at E7.5 and mice carrying a RING domain-disrupting MDMX mutant die at E9.5 (Itahana et al. 2007; Huang et al. 2011). Therefore, while mice expressing MDM2 I438K survive longer than other MDM2 E3-deficient mice, the ability of MDM2 I438K to restrain p53 activity is not sufficiently robust to support full embryonic development.

To confirm that the observed embryonic lethality in *Mdm2*^I438K/I438K^ mice was a consequence of unchecked p53 activity during development, we generated a compound MDM2 I438K and p53 knockout mouse (*Mdm2*^I438K/I438K^; *p53*^KO/KO^) through intercrossing *Mdm2*^I438K/WT^; *p53*^KO/WT^ mice. Consistent with the reports from other MDM2-null and MDM2 E3-deficient mice, *Mdm2*^I438K/I438K^; *p53*^KO/KO^ mice were viable and indistinguishable from littermate control mice (*Mdm2*^I438K/WT^; *p53*^KO/KO^) (Supplemental Fig. S2B,C; Jones et al. 1995; Montes de Oca Luna et al. 1995; Chavez-Reyes et al. 2003; Itahana et al. 2007). Tissues from *Mdm2*^I438K/I438K^; *p53*^KO/KO^ and *Mdm2*^I438K/WT^; *p53*^KO/KO^ mice at 5 mo of age exhibited similar organ morphology and histological analysis revealed that both sets of mice had developed small to medium-sized thymic lymphomas independently of MDM2 status by this time (Supplemental Fig. S2B), consistent with the normal disease trajectory of *p53*^KO/KO^ mice (Donehower et al. 1995).

Given the lethality of *Mdm2*^I438K/I438K^ constitutive mice, we sought to examine the consequences of switching to MDM2 I438K expression in adult mice by using *Rosa*-Cre^ER^ to replace *Mdm2* WT exons 11/12 with those containing the mutant *Mdm2*^I438K^ allele (tm1), through tamoxifen-induced Cre-mediated recombination. An initial induction protocol using the intraperitoneal (IP) injection of tamoxifen resulted in progressive weight loss in *Mdm2*^I438K/I438K^; *Rosa*-Cre^ER^-positive mice that reached clinical endpoint (Fig. 2C). At necropsy, these mice had diarrhea and subsequent histological examination revealed clear damage to the colon and small intestine in IP tamoxifen-induced *Mdm2*^I438K/I438K^; *Rosa*-Cre^ER^-positive mice (Fig. 2D; Supplemental Fig. S2D). Along with the sustained weight loss, our observations suggested gut toxicity arising as a consequence of our induction regime. We hypothesized that this outcome might reflect an on-target p53 response to intestinal stress caused by the IP delivery of a bolus of sunflower oil, the vehicle for tamoxifen in this experiment. To test this idea, we induced *Mdm2*^I438K/I438K^; *Rosa*-Cre^ER^-positive mice through the subcutaneous (SC) delivery of tamoxifen instead, after having first validated this method by assessing recombination using *R26*^tdRFP^; *Rosa*-Cre^ER^ reporter mice (Luche et al. 2007). In the *R26*^tdRFP^ mouse, Cre recombinase activity excises the Lox-STOP-Lox (LSL) cassette blocking ubiquitous *Rosa26*-linked RFP expression (R26^LSL-tdRFP^), allowing for RFP expression only in tissues that have undergone successful recombination. As such, analysis for RFP reveals where Cre recombinase was active in each reporter mouse (Luche et al. 2007). In *R26*^tdRFP^; *Rosa*-Cre^ER^ mice, we observed equivalent RFP expression by IHC across all tissues examined between mice induced by tamoxifen given through IP or SC injection, confirming equivalent induction efficiency with the SC induction regime (Supplemental Fig. S2E,F).

Using the SC tamoxifen induction regime, there was no detectible deleterious effect of the switch to homozygous MDM2 I438K expression on the weight or activity of *Rosa*Cre^ER^; *Mdm2*^I438K/I438K^ mice over the first 10 d following tamoxifen treatment (Fig. 2E). By comparison, deletion of MDM2 in adult *Mdm2*^FL/FL^ mice using the same SC tamoxifen protocol resulted in rapid weight loss by 4 d after the first tamoxifen injection (Fig. 2E) – results that are in accordance with previously published data (Ringshausen et al. 2006; Zhang et al. 2014). The rapid response to *Mdm2* deletion using this Cre induction protocol indicates efficient recombination of the *Mdm2* allele, comparable with that seen in previous studies. Further analysis of liver and small intestine from the induced *Rosa*Cre^ER^; *Mdm2*^I438K/I438K^ mice at 5 and 10 d after induction confirmed efficient recombination of the mutant allele (Supplemental Fig. S2G), suggesting a lack of short-term selection against MDM2 I438K expression. Histological examination of *Rosa*Cre^ER^; *Mdm2*^I438K/I438K^ mice revealed no pronounced changes in tissue histology after either 5 or 10 d after the switch to MDM2 I438K expression (Fig. 2F,G). Indeed, the MDM2 I438K-expressing mice remained healthy for at least 4 mo and were indistinguishable from littermates throughout this timeframe in terms of survival, weight gain, and activity (Fig. 2H,I). Taken together, the data suggest that MDM2 I438K is capable of limiting p53 activity in adult mice in vivo.

### Organ-specific changes in p53 activity following MDM2 I438K induction

**Figure 3.**
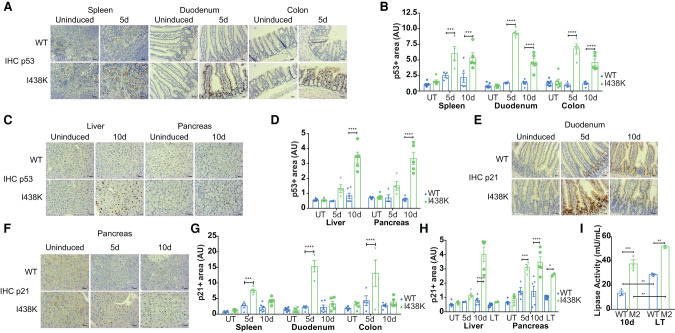
Persistent p21 expression corresponds with organ dysfunction after switch to *Mdm2*^*I438K*^ expression in vivo. (*A*–*D*) Representative IHC staining and quantification of p53 intensity in the indicated proliferative (*A*,*B*) and nonproliferative (*C*,*D*) tissues of *Rosa*Cre^ER^: *Mdm2*^I438K/I438K^ (I438K) and *Rosa*Cre^ER^; *Mdm2*^WT/WT^ (WT) animals at indicated time points following initial dose of subcutaneous (SC) tamoxifen induction. Scale bar, 20 μm. *N* = 7 WT and *N* = 6 I438K uninduced mice (UT). *N* = 4 mice per group at 5 d after induction and *N* = 6 WT and *N* = 5 I438K mice at 10 d after induction. Data are presented as mean ± SEM and analyzed using two-way ANOVA with Holm-Sidak's multiple comparisons test and multiplicity-adjusted *P*-values examining effects of genotype over time within each tissue. (*E*–*H*) Representative IHC staining in the duodenum (*E*) and pancreas (*F*) and quantification of p21 intensity in the indicated proliferative (*G*) and nonproliferative (*H*) tissues of the same mice as *A*–*D* at indicated time points following initial dose of tamoxifen induction (SC). The p21 data in *H* additionally include quantification of p21 in the liver and pancreas of mice in the longer term (2 and 4 mo) after induction (LT). *N* = 3 WT and *N* = 2 I438K LT mice. Scale bar, 20 μm. Data presented as mean ± SEM and analyzed using two-way ANOVA with Holm-Sidak's multiple comparisons test and multiplicity-adjusted *P*-values examining effects of genotype over time within each tissue. (*I*) Determination of plasma lipase activity (milliunits per milliliter) from *Rosa*Cre^ER^; *Mdm2*^I438K/I438K^ (M2) and *Rosa*Cre^ER^; *Mdm2*^WT/WT^ (WT) animals 10 d after initial dose of SC tamoxifen induction (10 d) and in the longer term (2 and 4 mo) after induction (LT). *N* = 3/group except *N* = 2 LT *Mdm2*^I438K/I438K^ mice. Mean ± SEM and data points shown. Data for 10-d mice are also shown in Figure 5K (“no IR”) and data from LT mice are also shown in Figure 4K. Data analyzed using two-way ANOVA with Holm-Sidak's multiple comparisons test and multiplicity-adjusted *P*-values.

Immunohistochemical analysis of *Rosa*Cre^ER^; *Mdm2*^I438K/I438K^ mice at 5 and 10 d after SC tamoxifen induction showed a clear stabilization of p53 across all organs tested, compared with mice expressing wild-type MDM2 (*Rosa*Cre^ER^; *Mdm2*^WT/WT^) (Fig. 3A–D). However, the time to reach peak stabilization of p53 varied across tissues, with high-levels of p53 evident by 5 d after the first tamoxifen injection in proliferative tissues—including the small intestine, colon, and spleen—and a more gradual accumulation over 10 d in the pancreas and liver (Fig. 3A–D).

To assess the ability of MDM2 I438K to regulate p53 activity, we examined p21 protein expression by IHC in various organs of these mice at 5 and 10 d after induction. In contrast to cells in culture, where p21 expression remained low despite p53 accumulation, each tissue showed an induction of p21 protein that coincided with the stabilization of p53 (Fig. 3E–H). Specifically, p21 accumulation was highest in the spleen, duodenum, and colon at 5 d after induction, with evidence for a reduction (spleen) or a return to baseline (duodenum and colon) expression by 10 d, despite the maintenance of high p53 levels at this time point (Fig. 3B). In the liver and pancreas, an increase in p21 levels was delayed, with maximal induction seen at 10 d after induction, coinciding with the delayed accumulation of p53 (Fig. 3H). Two important responses to p53 activation are proliferative arrest and induction of cell death. While p21 is a key mediator of p53-induced cell cycle arrest, p53 activates the expression several apoptotic proteins, including BAX. Interestingly, BAX expression following induction of MDM2 I438K generally followed the pattern of p21 expression (Supplemental Fig. S3A,B), with a transient increase at 5 d after induction that declined by 10 d in the proliferative organs (spleen, duodenum, and colon) and a persistence of expression at 10 d in liver and pancreas. Further analysis of duodenum and liver confirmed the pattern of *Cdkn1a* (*p21)* and *Bax* expression at the mRNA level (Supplemental Fig. S3C,D), consistent with this being a transcriptional response to p53. Furthermore, the expression of *Noxa* (also known *Pmaip1*) and *Puma* (also known as *Bbc3*), additional apoptotic p53 target genes, generally confirmed this pattern of expression (Supplemental Fig. 3SC,D). The exception was *Puma* mRNA expression in the liver, which increased at 5 d, but then declined to baseline by 10 d after induction (Supplemental Fig. S3D).

Taken together, these observations suggest that although the MDM2 I438K mutant is able to restrain p53 levels sufficiently to prevent weight loss or lethality, the mutant does not completely limit the transcriptional activity of stabilized p53 in all murine tissues. To determine the outcome to these responses, we tested proliferation (measured by Ki-67 staining) and apoptosis (measured by CC3 staining). In most tissues, despite the induction of expression of p21 and several apoptotic proteins, there was no evidence of significant proliferative arrest or apoptosis following induction of MDM2 I438K (Supplemental Fig. S3E–G), although a transient reduction in proliferation was seen in the spleen at 5 d, which recovered by 10 d. Overall, these data suggest that the increased p53 activity in *Rosa*Cre^ER^; *Mdm2*^I438K/I438K^ mice was not sufficient to cause undue arrest or cell death, consistent with the lack of toxicity in mice following induction.

Despite this lack of evidence for either proliferative arrest or apoptosis, our data revealed a persistence of the p53 response in liver and pancreas at 10 d after induction and additional analysis of p21 expression in the longer term following induction showed a recovery to baseline in the liver after several months but persistence of enhanced expression in the pancreas (Fig. 3H). To examine more closely whether there were any consequences of induction of the p53 response in *Rosa*Cre^ER^; *Mdm2*^I438K/I438K^ mice, we analyzed liver and pancreas function in both 10 d after induction mice and longer-term age-induced mice. Liver function was assessed by measuring plasma activity of ALT (alanine aminotransferase) and AST (aspartate aminotransferase), two liver enzymes that are elevated in the circulation following liver damage. Modestly increased levels of both ALT and AST activity were seen in the plasma of 10-d induced *Rosa*Cre^ER^; *Mdm2*^I438K/I438K^ mice compared with control mice (Supplemental Fig. S3H,I), although these levels normalized over the longer term, suggesting that any liver dysfunction was mild and transient and consistent with the recovery of restrained p21 expression by this time. To assess pancreas function, we examined the levels of plasma lipase, a pancreatic enzyme that is increased in the blood following pancreatic damage. Interestingly, plasma lipase activity increased with age in the wild type mice (Fig. 3I). However, we also observed significantly increased levels of plasma lipase in 10-d induced *Rosa*Cre^ER^; *Mdm2*^I438K/I438K^ mice that persisted in the longer term, suggesting an ongoing but stable increase in pancreatic dysfunction resulting from persistent expression of the MDM2 I438K mutant (Fig. 3I).

### Rapid induction of the p53 stress response in MDM2 I438K-expressing mice

**Figure 4.**
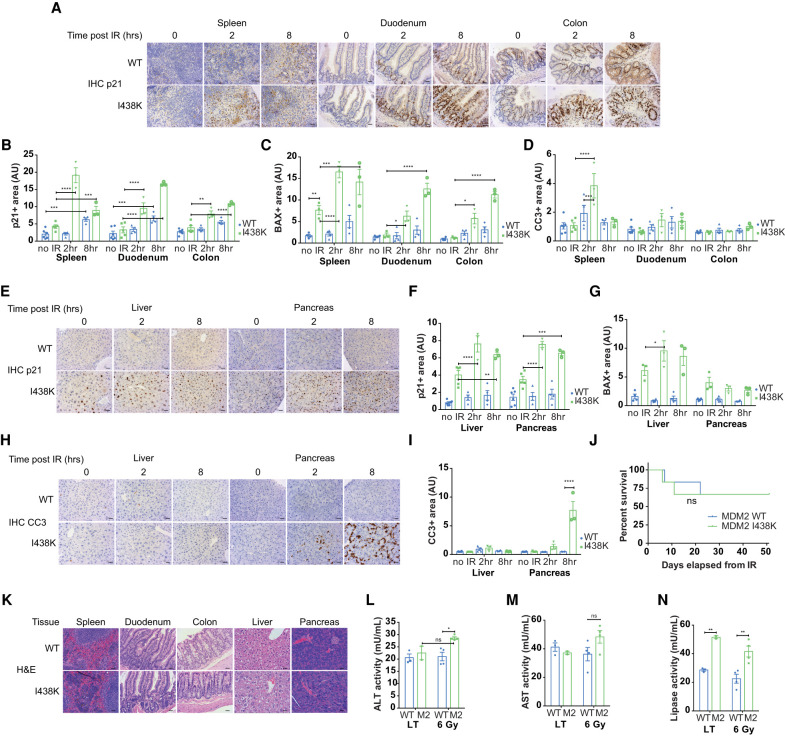
*Mdm2*^I438K^ mice respond more robustly to 6 Gy IR, leading to acute lethality in the pancreas. (*A*) Representative IHC staining for p21 in the indicated proliferative tissues at the indicated time points (in hours) after 6 Gy TBI administered 10 d after SC tamoxifen induction in *Rosa*Cre^ER^; *Mdm2*^I438K/I438K^ (I438K) and *Rosa*Cre^ER^; *Mdm2*^WT/WT^ (WT) mice. *N* = 6 WT and *N* = 5 I438K control induced mice (no IR) and *N* = 4 WT and *N* = 3 I438K mice per irradiation time point. Scale bar, 20 μm. (*B*–*D*) Quantification of p21 (*B*), BAX (*C*), and cleaved caspase 3 (CC3; *D*) staining in the indicated proliferative tissues of the mice from *A*. Data are presented as mean ± SEM and analyzed using two-way ANOVA with Holm-Sidak's multiple comparisons test and multiplicity-adjusted *P*-values examining effects of genotype over time within each tissue. (*E*) Representative IHC staining for p21 in the liver and pancreas at the indicated time points (in hours) after 6 Gy TBI administered 10 d after SC tamoxifen induction in *Rosa*Cre^ER^; *Mdm2*^I438K/I438K^ (I438K) and *Rosa*Cre^ER^; *Mdm2*^WT/WT^ (WT) mice. *N* = 6 WT and *N* = 5 I438K control induced mice (no IR) and *N* = 4 WT and *N* = 3 I438K mice per irradiation time point. Scale bar, 20 μm. (*F*,*G*) Quantification of p21 (*F*) and BAX (*G*) staining in the indicated nonproliferative tissues of the mice depicted in *A*. Data are presented as mean ± SEM and analyzed using two-way ANOVA with Holm-Sidak's multiple comparisons test and multiplicity-adjusted *P*-values examining effects of genotype over time within each tissue. (*H*,*I*) Representative IHC staining and quantification of cleaved Caspase 3 (CC3) in the liver and pancreas of mice from *E*. Data presented as mean ± SEM and analyzed using two-way ANOVA with Holm-Sidak's multiple comparisons test and multiplicity-adjusted *P*-values examining effects of genotype over time within each tissue. (*J*) Survival curve comparing adult *Rosa*Cre^ER^; *Mdm2*^I438K/I438K^ (I438K) and *Rosa*Cre^ER^; *Mdm2*^WT/WT^ (WT) mice for 50 d after 6 Gy TBI treatment. *N* = 6 mice/group. *N* = 2 mice/group reached clinical endpoint prior to 50 d. Survival analyzed using Log-rank (Mantel-Cox) test. (ns) Not significant. (*K*) Representative H&E staining of the indicated tissues in *Rosa*Cre^ER^; *Mdm2*^I438K/I438K^ (I438K) and *Rosa*Cre^ER^; *Mdm2*^WT/WT^ (WT) mice at 50 d after 6 Gy TBI. *N* = 4 mice/group. Scale bar, 20 μm. (*L–N*) Determination of plasma ALT (*L*), AST (*M*), and lipase activity (*N*) (all in milliunits per milliliter) in *Rosa*Cre^ER^; *Mdm2*^I438K/I438K^ (M2) and *Rosa*Cre^ER^; *Mdm2*^WT/WT^ (WT) mice in the longer term (2 and 4 mo) after induction (LT) and mice at 50 d after 6 Gy TBI (6 Gy). *N* = 4 mice/group in 50-d cohorts, *N* = 3 LT WT mice, and *N* = 2 LT *Mdm2*^I438K/I438K^ mice. Mean ± SEM and data points shown. Data analyzed using two-way ANOVA with Holm-Sidak's multiple comparisons test and multiplicity-adjusted *P*-values. Data from aged mice are also presented in Supplemental Figure S3, H and I (ALT/AST), and Figure 3I (lipase).

In our in vitro experiments, *Mdm2*^I438K^-expressing cells proliferated normally but were significantly more sensitive to p53 activating signals such as Nutlin and doxorubicin (Fig. 1; Supplemental Fig. S1). To determine whether the same enhanced response to stress could be detected in vivo, we exposed pairs of 10-d postinduction MDM2 WT and *Rosa*Cre^ER^; *Mdm2*^I438K/I438K^ mice to nonlethal 6 Gy total body γ-irradiation (TBI) and followed the p53 response over a short 8-h time course. Consistent with previous reports (Pant et al. 2013; Tollini et al. 2014), these studies revealed stabilization of p53 in the radiosensitive spleen, duodenum, and colon after TBI in MDM2 WT mice (Supplemental Fig. S4A) with a corresponding increase in p21 protein expression by 8 h after TBI (Fig. 4A,B). In *Rosa*Cre^ER^; *Mdm2*^I438K/I438K^ mice, p53 levels were higher before TBI and showed a further rapid stabilization by 2 h (Supplemental Fig. S4A), correlating with a more robust induction of p21 in these tissues compared with MDM2 WT mice (Fig. 4A,B). Interestingly, evidence for a rapid recovery from this response was seen in the spleen where p53 and p21 levels declined by 8 h after TBI. Levels of BAX protein were similarly induced to higher levels in I438K spleen, duodenum and colon than in WT tissue (Fig. 4C), a response that was more pronounced in the spleen and corresponded with increased cell death in spleen, but not the duodenum or colon at these early time points (Fig. 4D). Analysis of mRNA expression in duodenum confirmed increased *Cdkn1a* (*p21)*, *Bax*, *Noxa*, and *Puma* expression in response to TBI in MDM2 WT mice with a substantial enhancement of this response for all p53 target genes in the *Rosa*Cre^ER^; *Mdm2*^I438K/I438K^ mice (Supplemental Fig. S4B). However, despite the increase in apoptotic gene expression, only the spleen showed transient elevation in apoptosis at 2 h after TBI (Fig. 4D).

In contrast to radiosensitive tissues, no clear stabilization of p53 was detected in radioresistant tissues (pancreas and liver) in response to 6 Gy TBI in WT mice, although in I438K mice both tissues sustained higher levels of p53 (Supplemental Fig. S4C). Similarly, p21 and BAX levels were substantially higher in I438K liver and pancreas both before and after TBI (Fig. 4E–G). Transcription of *Cdkn1a* (*p21)*, *Bax* and *Noxa* was also significantly higher in the liver of TBI I438K mice compared with wild-type mice (Supplemental Fig. S4D), although liver *Puma* transcription was not increased in either wild-type or I438K mice in response to IR, supporting some tissue specificity to the p53 targets that are engaged in response to stress. Interestingly, while no cell death was apparent in the liver, the pancreas showed a rapid and dramatic increase in CC3 staining within MDM2 I438K-expressing acinar cells (Fig. 4H,I).

The increase in the strength and duration of the activation of the p53 response in *Rosa*Cre^ER^; *Mdm2*^I438K/I438K^ mice following acute nonlethal TBI, and especially the unexpectedly potent engagement of p53-induced cell death in the pancreas, raised the possibility of more severe long-term consequences of exposure to p53 activating signals in *Rosa*Cre^ER^; *Mdm2*^I438K/I438K^ mice. Previous studies have shown that while 6 Gy TBI can be tolerated by *Mdm2* WT mice without impact on medium-term survival, it can be lethal in a mouse model where the MDM2-p53 feedback loop is removed (MDM2 P2 mice) or in mice with an extreme C-terminal *Mdm2* mutation that disrupts MDM2 E3 activity (Pant et al. 2013; Tollini et al. 2014). However, we found no decreased survival in *Rosa*Cre^ER^; *Mdm2*^I438K/I438K^ mice exposed to 6 Gy TBI compared with *Mdm2* WT mice (Fig. 4J). At 50 d after 6 Gy TBI, *Rosa*Cre^ER^; *Mdm2*^I438K/I438K^ and *Rosa*Cre^ER^; *Mdm2* WT mice showed similar tissue histology (Fig. 4K), although we did observe slightly elevated plasma ALT and AST activity—suggesting some liver dysfunction arising from 6 Gy TBI (Fig. 4L,M). Interestingly, the acute pancreatic acinar attrition observed in *Rosa*Cre^ER^; *Mdm2*^I438K/I438K^ mice after 6 Gy TBI was not accompanied by a further loss of pancreas function–seen as a maintenance but not increase in plasma lipase activity (Fig. 4N). These observations suggest some level of recovery and regeneration of the exocrine pancreas (Zhou and Melton 2018).

**Figure 5.**
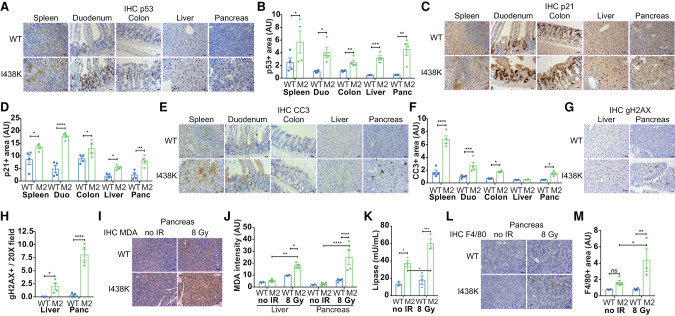
The acute response to lethal 8 Gy IR is exacerbated in both sensitive and nonradiosensitive tissues in *Mdm2*^I438K^ mice. (*A*,*B*) Representative IHC staining and quantification of p53 in the indicated tissues at 3 d after 8 Gy TBI treatment administered 10 d after beginning of SC tamoxifen induction in *Rosa*Cre^ER^; *Mdm2*^I438K/I438K^ (M2) and *Rosa*Cre^ER^; *Mdm2*^WT/WT^ (WT) mice. *N* = 4 mice/group. Scale bar, 20 μm. (Duo) Duodenum, (Panc) pancreas. Data analyzed using multiple *t*-tests with Holm-Sidak's method to correct for multiple comparisons. (*C*,*D*) Representative IHC staining and quantification of p21 as in *A* and *B*. *N* = 4 mice/group. (*E*,*F*) Representative IHC staining and quantification of cleaved caspase 3 (CC3) as in *A* and *B*. *N* = 4 mice/group. (*G*,*H*) Representative IHC staining and quantification of phospho-Histone H2A.X (Ser139) (gH2AX) in the liver and pancreas at 3 d after 8 Gy TBI treatment administered 10 d after beginning of SC tamoxifen induction in *Rosa*Cre^ER^; *Mdm2*^I438K/I438K^ (M2) and *Rosa*Cre^ER^; *Mdm2*^WT/WT^ (WT) mice. *N* = 4 mice/group. Scale bar, 20 μm. Data analyzed using multiple *t*-tests with Holm-Sidak's method to correct for multiple comparisons. (*I*,*J*) Representative IHC staining and quantification of malondialdehyde (MDA) as in *G* and *H*. *N* = 4 mice/group. Scale bar, 20 μm. Data analyzed using two-way ANOVA with Holm-Sidak's multiple comparisons test and multiplicity-adjusted *P*-values. (*K*) Comparison of plasma lipase activity (milliunits per milliliter) in *Rosa*Cre^ER^; *Mdm2*^I438K/I438K^ (M2) and *Rosa*Cre^ER^; *Mdm2*^WT/WT^ (WT) mice at 10 d after the start of induction (no IR) and at 3 d after 8 Gy TBI treatment administered 10 d after beginning of tamoxifen induction. *N* = 3 mice/group. Data from “no IR” mice also shown in Figure 3I (“young induced” mice). Data analyzed by two-way ANOVA with Holm-Sidak's multiple comparisons test and multiplicity-adjusted *P*-values. (*L*,*M*) Representative IHC staining and quantification of F4/80 in the pancreas as in *G* and *H*. *N* = 4 WT mice and *N* = 3 M2 mice. Scale bar, 20 μm. Data analyzed using two-way ANOVA with Holm-Sidak's multiple comparisons test and multiplicity-adjusted *P*-values.

In humans, targeted radiation treatment for cancer patients can result in exposure of both tumor-adjacent normal and more distant peripheral tissues to a lethal irradiation dose (Quast 2006). Given the well-documented on-target toxicities limiting the therapeutic efficacy of “Nutlin-like” compounds, we were interested in exploring the effect of putative MDM2 E3 inhibitors on normal tissue in response to higher radiation exposure. We chose 8 Gy TBI, a dose on the low end of the lethal range for mice, with an expected LD50 time (time until lethal dose for 50% of mice) beyond 1 wk (Booth et al. 2012), combined with an early sampling time (3 d after TBI). At 3 d after 8 Gy TBI, mice were similarly active in each cohort and did not yet exhibit clinical signs of radiation sickness. Histology of the radiosensitive spleen, duodenum, and colon was similar in *Rosa*Cre^ER^; *Mdm2*^I438K/I438K^ and *Rosa*Cre^ER^; *Mdm2* WT mice (Supplemental Fig. S5A), although as expected, the *Rosa*Cre^ER^; *Mdm2*^I438K/I438K^ mice exhibited increased levels of both p53 and p21 staining (Fig. 5A–D). However, in contrast to our data from short-term 6 Gy TBI treatment, the enhanced p53 signaling that we observed in *Rosa*Cre^ER^; *Mdm2*^I438K/I438K^ mice after 8 Gy TBI treatment correlated with increased CC3 staining across all radiosensitive tissues (Fig. 5E,F).

As in radiosensitive organs, we noted robust p53 accumulation and p21 expression in both the liver and the pancreas of *Rosa*Cre^ER^; *Mdm2*^I438K/I438K^ mice (Fig. 5A–D), although increased CC3 staining was only evident in the pancreas and not in the liver (Fig. 5E,F). Both the liver and pancreas showed evidence of deteriorated tissue architecture and unresolved DNA damage, identified by phospho-H2AX-positive foci (gH2AX) (Fig. 5G,H), and increased cellular ROS (Fig. 5I,J; Supplemental Fig. S5B). However, liver function in *Rosa*Cre^ER^; *Mdm2*^I438K/I438K^ mice, measured by plasma ALT and AST activity, was not further compromised by 8 Gy TBI treatment at this time point (Supplemental Fig. S5C,D). In contrast, pancreas function, as judged by plasma lipase activity, showed further deterioration following 8 Gy TBI (Fig. 5K). Further evidence for increased pancreas damage was provided by the identification of greater macrophage content, as judged by F4/80 staining, in the pancreata of I438K mice (Fig. 5L,M), reminiscent of the increased macrophage infiltration evident during pancreatitis (Habtezion 2015; Xue et al. 2015).

## Discussion

We have described the in vivo characterization of an E3 ubiquitin ligase-deficient MDM2 mutant, MDM2 I438K, which lacks the ability to interact with E2/ubiquitin but maintains an otherwise functional MDM2 RING finger domain. Although the MDM2 I438K mutant has lost the E3-dependent features of MDM2 regulation of p53, it retains the ability to control p53 transcriptional activity. Several MDM2 mutant mice have been reported, including those altered in the RING domain (C462A) and in the C-terminal tail (Y487A) (Itahana et al. 2007; Tollini et al. 2014). Unlike the I438K mutant described here, C462A disrupts the structure of the RING and results in more general loss of the ability to control p53. The C-terminal tail Y487A mutant, by comparison, compromises MDM2 homodimer binding to E2-Ub, but can be rescued by heterodimerization with MDMX. A clear difference between the efficiency of Y487A and I438K in controlling p53 is seen by the embryonic lethality associated with the expression of the latter, but not the former. Our data indicate that the E3 ligase activity of MDM2 is necessary for embryogenesis.

In contrast, induction of I438K expression in the adult mouse was not lethal and these mice showed no overt pathologies compared with wild-type mice over the course of several months. As seen in vitro, p53 accumulated in the tissues of mice expressing MDM2 I438K, consistent with the loss of E3 activity. However, unlike the in vitro models, this accumulation of p53 provoked the induction of a p53 response, as measured by an increase in p21 expression. This activation of p53 was transient in radiosensitive tissue and was only seen to persist in the pancreas. There appear to be tissue-dependent differences in the response to loss of MDM2 E3 activity and the ability to regain homeostasis of the control of p53. These findings suggest the existence of cell-specific factors that either limit or promote MDM2 I438K efficacy in controlling p53 in vivo that are not recapitulated in in vitro cell culture systems.

Importantly, while the partial control of p53 activity shown by MDM2 I438K in vivo is sufficient to allow for normal growth, these mice displayed an enhanced p53 response to DNA damage-inducing TBI. Under nonlethal TBI, increased p53 activity—as indicated by increased expression of p21—was detected in radiosensitive tissues, but this did not lead to increased cell death or decreased survival. Intriguingly, the MDM2 tail region mutant Y487A expressing mice were highly sensitive to an even lower dose of TBI (5 Gy) and showed 100% mortality within 20 d of treatment (Tollini et al. 2014), accompanied by haematopoietic failure and apoptosis in the spleen. Furthermore, treatment of *Rosa*Cre^ER^; *Mdm2*^I438K/I438K^ mice with a lethal dose of TBI did not reveal an overt increase in toxicity, although again evidence of enhanced stabilization and activation of p53 was seen in various organs.

An intriguing exception to the general tolerability of MDM2 I438K expression was the pancreas, where expression of MDM2 I438K sensitized acinar cells to p53 apoptotic signaling. Even at baseline, the pancreata in *Rosa*Cre^ER^; *Mdm2*^I438K/I438K^ mice exhibited features of chronic pancreatic dysfunction as judged by elevated plasma lipase. Beyond that, a mild applied stress such as 6 Gy TBI—irradiation that caused no discernible changes to MDM2 WT pancreata—was sufficient to cause rapid and widespread acinar attrition in *Rosa*Cre^ER^; *Mdm2*^I438K/I438K^ mice. Perhaps due to the impressive regenerative capacity of the exocrine pancreas, both the baseline pancreatic stress and the acute 6 Gy TBI-induced acinar cell death appeared ultimately tolerable and did not compromise the viability of aging *Rosa*Cre^ER^; *Mdm2*^I438K/I438K^ mice or mice in the medium term after 6 Gy TBI.

While we show persistence of the recombined MDM2 allele over the short term, we cannot exclude the selection of nonrecombined wild-type MDM2 expressing cells in the long-term aged mice. However, the maintenance of elevated p21 in the pancreas suggests the retention of some level of p53 response consistent with MDM2 I438K expression. Despite the maintenance of murine viability, evidence of persistent pancreas damage was observed. Furthermore, treatment of *Rosa*Cre^ER^; *Mdm2*^I438K/I438K^ mice with lethal 8 Gy TBI caused even greater pancreatic damage, including high levels of unresolved DNA damage, increased cell death, increased cellular ROS, macrophage infiltration and further compromised pancreatic function. Although these features were not universally translated to the liver, a second traditionally nonradiosensitive organ, even here the *Rosa*Cre^ER^; *Mdm2*^I438K/I438K^ mice exhibited elevated DNA damage and higher levels of ROS after 8 Gy TBI. These findings suggest a mixture of tissue-specific and generalizable features of MDM2 I438K function that can exacerbate the p53 response to genotoxic damage.

Taken together, our findings suggest that the E3-deficient MDM2 I438K mutant provides enough control over elevated p53 levels to allow for homeostasis in adult mice. Expression of this mutant significantly enhanced the p53 response in these animals, although this was not associated with decreased survival in the face of TBI. More broadly, our results examining the MDM2 I438K mutant can be viewed as a genetic approximation of the potential efficacy and utility of future small molecule inhibitors specifically targeting MDM2 E3 activity rather than the MDM2-p53 interaction and act as a proof of principle for this approach. In this light, our findings suggest that such small molecule inhibitors would not show toxicity in many normal tissues and may be more likely to activate an on-target p53 response in stressed preneoplastic tissues and tumors. More specifically, an E3 inhibitor may have wide-ranging and tissue-specific therapeutic windows based on the underlying sensitization of each normal tissue to p53-mediated cell death from genotoxic combination therapies. Based on our evidence, liver cancers that retain wild-type p53, for example, may be more likely to usefully respond to MDM2 E3 inhibition than pancreatic cancers, where the sensitivity to on-target toxicity in acinar cells from genotoxic combination therapy may be high.

## Materials and methods

### Generation of *Mdm2*^I438K^ knock-in mice

A conditional point mutant allele of the *Mdm2* gene (Ensembl ID: ENSMUSG00000020184 in Genome Assembly GRCm38.p6) was generated by gene targeting. This allele replaces exons 11 and 12 (ENSMUSE00001296149 & ENSMUSE00000665547) of the mouse *Mdm2* gene transcript (Ensembl transcript ID: Mdm2-201; ENSMUST00000020408.15) with an alternate version of exons 11 and 12 encoding the I438K point mutation upon Cre recombination. This changes the ATC codon at positions 418–420 of *Mdm2* exon12 to AAG, replacing the isoleucine at position 438 with lysine.

An F3-Neomycin-R cassette was inserted into the *Mdm2* targeting vector by cotransfection of EL250 *E. coli* (Liu et al. 2003). These *E.coli* can express Flp recombinase under arabinose induction. The DNA fragment flanked by the homology arms was excised and recombineered (Liu et al. 2003) into a pool of mouse genomic DNA BAC clones (Source Biosciences) carrying the mouse *Mdm2* gene in EL350 *E.coli* (Adams et al. 2005). A retrieval plasmid was generated by the PCR amplification of NotI-AscI linearized pFlexDTA (a modified version of pFlexible; [van der Weyden et al. 2005]). The modified sequences were retrieved from BAC clones in EL250 *E.coli* by recombineering.

The targeting vector was sequence verified prior to transfection. It was then linearized by AscI digestion and used to transfect HM1 mESCs by electroporation (Magin et al. 1992). Cells were selected under neomycin (300 µg/mL) and surviving colonies were picked and screened for targeting by long range PCR (using the Roche Expand long template PCR system) from within the neomycin-resistance cassette to sequences beyond the ends of the homology arms. Oligo sequences used to screen cells to ensure appropriate targeting of the *Mdm2* gene were GTGGATTGATTTAGGAAACAAGATG and CAAGTTAACAACAACAATTGCATTC for the 5′ side and GCATTGTCTGAGTAGGTGTCATTC and ACGCAACATTAATACAAAGCTATCC for the 3′ side.

Following identification of correctly targeted clones, mouse lines were derived by injection of ES cells into C57BL/6J blastocysts according to standard protocols (Nagy et al. 2003). After breeding of chimeras, germline offspring were identified by coat color and the presence of the modified allele was confirmed with the primers described above. The point mutation and recombination sites were sequence verified in the mice. Mice carrying the tm1.1 allele were generated by crossing mice harboring the tm1 allele with a mouse line expressing Flpe (Tg(ACTFLPe)9205Dym) to delete the selectable marker by recombination at the FRT sites (Rodríguez et al. 2000). Deletion of the selectable marker was confirmed by PCR across the remaining F3 site.

### Mice

*Rosa*-Cre^ER^ [Gt(ROSA)26Sor^tm2(cre/ERT2)Brn^], Deleter-Cre [Tg^(CMV-cre)1Cgn^], RFP reporter [Gt(ROSA)26Sor^tm1Hjf^], *p53*^KO/KO^ (*Trp53*^tm1Brd^), *Mdm2*^FL/FL^ (*Mdm2*^tm2.1Glo^), and Flippase recombinase [Tg^(ACTFLPe)9205Dym^] mice were described previously (Donehower et al. 1992; Schwenk et al. 1995; Rodríguez et al. 2000; Grier et al. 2002; Hameyer et al. 2007; Luche et al. 2007). The induction time-course, 6 Gy TBI short time-course, and medium-term 6 Gy TBI 50-d mice were a mix of male and female mice. Longer-term aging mice were all male. The acute 3-d 8 Gy TBI treated mice were all female. Mice within each experiment were age and littermate matched, and all induced at the same time to the extent this was possible. Mice were treated with tamoxifen prior to 6 mo of age. Downstream analyses were performed on a random order of samples blinded to the genotype and treatment regime until the summation of results.

Procedures involving mice were performed under Home Office licence numbers 70/8645 and 60/4293. Experiments were conducted in accordance with the Animals (Scientific Procedures) Act 1986 and the EU Directive 2010 and sanctioned by Local Ethical Review Process (University of Glasgow). Mice were housed on a 12-h light/12-h dark cycle and provided with normal chow diet and water ad libitum. All experimental cohort mice were genotyped by Transnetyx. Founding mice, BMK cells, and MEF cell lines were genotyped by hand using the genotyping PCR protocols outlined below.

Tamoxifen (Sigma T5648) was administered to mice >20 g via intraperitoneal injection a total of four times (3 mg of tamoxifen on day 1, followed by 2 mg of tamoxifen on days 2, 3, and 4) or by subcutaneous (SC) injection a total of four times (3 mg of tamoxifen on day 1, followed by 2 mg of tamoxifen on days 2, 3, and 4). This latter induction regime was well tolerated. However, a subset of male mice in our aged induced cohorts reached an unexpected early clinical end point at ∼60 d after induction due to unresolved hernias as has been previously described (Ma et al. 2015). This outcome was MDM2 and CreER genotype-independent.

For total body irradiation (TBI) experiments, mice were induced via the SC tamoxifen method and left for a total of 10 d from the first injection before proceeding with TBI. They were then treated with either 6 or 8 Gy TBI from an Xstrahl RS225 Cabinet X-ray Irradiator (Xstrahl) as indicated and sampled at the time points after TBI noted in the manuscript.

### Liver function assays (ALT/AST)

ALT and AST activity were determined in EDTA-treated plasma using the Alanine Transaminase Activity Assay Kit (ab105134) and the aspartate aminotransferase activity assay kit (ab138878) from Abcam. Both assays were performed following the manufacturer's recommendations. Samples were run together, analyzed in triplicate wells per mouse sample, and the mean value of these technical replicates was used for subsequent analysis.

### Pancreatic function assay (lipase)

Lipase activity was determined in EDTA-treated plasma using the lipase assay kit from Abcam (ab118969), according to the manufacturer's recommendations. Samples were run together, analyzed in triplicate wells per mouse sample, and the mean value of these technical replicates was used for subsequent analysis.

### Long-term mouse weights

Mice were weighed prior to the start of the SC tamoxifen induction regime and then periodically thereafter. During the induction protocol and shortly afterward, mice were weighed at least daily. Over the longer aging experiment, mice were monitored and weighed every other day. Mouse weights relative to the initial baseline were then plotted.

### Immunohistochemistry (IHC)

All IHC staining was performed on 4-μm formalin-fixed paraffin embedded (FFPE) sections that had previously been warmed for 2 h at 60°C. Slides were deparaffinized and rehydrated according to the relevant staining platform for each antibody as noted in the accompanying reagent and antibody information tables (Supplemental Tables S1,S2). Manual staining for MDA was performed as described previously (Humpton et al. 2018).

For staining on the Dako Autostainer Link 48 (Agilent), FFPE sections underwent manual dewaxing and rehydration through xylene and a graded ethanol series. Heat-induced epitope retrieval (HIER) was then performed on a Dako PT module where the sections were heated for 20 min to 97°C using target retrieval solution (TRS), high pH (Agilent). The sections underwent peroxidase blocking (Agilent) before application of primary antibody for 40 min at a previously optimized dilution. Secondary antibody incubation and downstream signal detection were performed using the rabbit envision secondary antibody for 30 min and liquid DAB (both Agilent).

For staining on the Leica Bond Rx autostainer, sections were loaded onto the autostainer and underwent dewaxing using Bond dewax solution and epitope retrieval using either ER2 buffer for 20 min at 95°C or enzyme 1 (Enz1) for 10 min at 37°C, as indicated (dewaxing and retrieval both done on-board; all reagents from Leica). Sections were rinsed with Leica wash buffer before undergoing peroxidase block for 5 min using an Intense R kit (Leica). Sections were incubated with a previously optimized concentration of primary antibody for 30 min. Secondary antibody incubation was performed using either the rabbit envision secondary antibody kit (Agilent) or Rat ImmPRESS secondary antibody kit (Vector labs) as appropriate for 30 min, and signal was visualized with DAB using the Intense R kit (Leica).

After detection in either system or manually, sections were counterstained with haematoxylin and coverslipped using DPX mountant (CellPath) prior to analysis.

### Analysis of IHC images

The analysis of IHC staining for all stains except phospho-H2AX was performed as previously described (Humpton et al. 2018). A minimum of five random nonoverlapping 20X magnification images were taken from each IHC slide using an Olympus BX51 microscope with Zen Blue software (Zeiss). From these images, the positive staining per slide was calculated using ImageJ software. For quantification of phospho-H2AX, positive acinar cells, or hepatocytes (pancreas/liver) per 20× field from the five images per mouse were quantified by hand and the mean value per mouse was reported in the relevant figures.

### Cell culture

MEF and BMK cells were generated as described below. Stock flasks of cells were maintained in DMEM high-glucose medium containing pyruvate (Gibco 21969035) supplemented with 2 mM L-glutamine, penicillin/streptomycin, gentamycin, and either 10% FBS (MEFs) or 5% FBS and ITS liquid medium supplement (BMK cells; Sigma Aldrich). Cells were cultured at 37°C in a humidified atmosphere of 5% CO_2_. Mycoplasma testing was not performed on these primary cell lines.

### BMK cells

BMK primary cells were isolated and cultured as previously described (Degenhardt et al. 2002). Briefly, the kidneys from 4 to 5-d-old baby mice were minced with 1.5 mL of collagenase (0.1 U/mL)/dispase (0.8 U/mL) solution and incubated for 40 min at 37°C. After incubation, single cells were isolated from the solution and cultured in DMEM supplemented with 5% FBS, 2 mM L-glutamine, penicillin/streptomycin, gentamycin, and ITS liquid medium supplement (Sigma I3146).

In all experiments with BMK cells, the expression of the *Mdm2*^I438K^ allele was induced by infection with recombinant adenovirus expressing empty vector control or Cre Recombinase for 24 h. After infection, the medium was changed and cells were left for 5 d. They were then treated with compounds (e.g., 4 μM Nutlin dissolved in DMSO for 8 h) and analyzed as indicated.

Recombinant adenovirus was purchased from the University of Iowa gene therapy core facility.

### MEFs

Primary MEFs were isolated as previously described from embryonic day E14.5 embryos (Humpton et al. 2019). The *p53* WT and *p53*^KO/KO^ MEFs used in Supplemental Figure S1 were generated from either *Arf^KO/KO^* mice (*p53* WT) or *p53^KO/KO^* mice and were provided by Daniel Murphy (*p53* WT) or Stephen Jones (*p53* KO). *p53*^KO/KO^; *Mdm2*^KO/KO^ double knockout MEFs in Supplemental Figure S1, E–G, were created from *p53*^KO/KO^; *Mdm2*^KO/KO^ mice and were provided by Stephen Jones. MEFs were cultured in DMEM supplemented with 10% FBS, 2 mM L-glutamine, penicillin/streptomycin and gentamycin.

In all experiments with the CreER-inducible MEFs, the expression of the *Mdm2*^I438K^ allele was induced by treatment with 1 μM 4-OHT dissolved in ethanol (Sigma H6278) for 48 h. After induction, the media was changed and cells were left for an additional 3 d. The MEFs were then treated with compounds (e.g., 4 μM Nutlin for 8 h) and analyzed as indicated.

### MDM2 and p53 plasmids

Mouse *p53* and mouse *Mdm2* pcDNA3.1 plasmids were previously described (Lee et al. 2015). The mCherry plasmid (pmCherry-C1) was also previously described (Werner et al. 1996). The mouse *Mdm2* mutant plasmids were generated from the WT mouse *Mdm2* plasmid using site-directed mutagenesis PCR as previously described (Nomura et al. 2017). The resulting *Mdm2* mutant plasmids were verified by DNA sequencing prior to use. Mouse FLAG-MDM4 was a gift from Jean-Christophe Marine. Plasmids are listed in Supplemental Table S3.

### p53 degradation assay

WT MEFs were transfected with the two pcDNA3.1 plasmids containing mouse *Trp53* and the indicated mouse *Mdm2* constructs, respectively, as well as the mCherry plasmid (pmCherry-C1), using GeneJuice (Novagen) following the manufacturer's recommendations. Briefly, a mixture of DNA plasmids (in a 1:1:1 ratio) and GeneJuice in OptiMEM was added dropwise to cell culture plates that had been seeded the day before. Reagent volumes, cell numbers, and DNA content were scaled according to plate size within the manufacture's recommended ranges. Cells were incubated for 24 h with the transfection mixture to allow for plasmid expression and then the degradation of p53 relative to mCherry was assessed through Western blot analysis.

### Cell cycle analysis

Previously induced BMK cells were treated with DMSO or H_2_O as vehicle control or with 4 μM Nutlin dissolved in DMSO (Sigma N6287) or 1 μM doxorubicin dissolved in H_2_O (Sigma 324380) for 8 h. The cells were then harvested with trypsin-EDTA, washed in cold PBS, and fixed in ice-cold 70% ethanol for at least 30 min at 4°C. Prior to analysis, cells were rehydrated with PBS and treated with 50 µg/mL RNase A in PBS for at least 15 min. DNA was stained with propidium iodide (PI) before flow cytometric analysis (Attune NxT, Thermo Fisher Scientific). DNA content was analyzed in channel FL2 and the percentage of cells in each phase was determined by DNA content analyzed by FlowJo 10 software (FlowJo) based on the Watson Pragmatic algorithm (Watson et al. 1987).

### Cell growth assay

MEFs were treated with 1 μM 4-OHT in medium for 48 h to induce expression of the *Mdm2*^I438K^ allele. The medium was replaced and MEFs were left for an additional 3 d. On day 5, MEFs were treated with DMSO or H_2_O as vehicle control or with 4 μM Nutlin or 1 μM doxorubicin for a period of 48 h. The medium was replaced with normal culture medium after 48 h. At the indicated time points (days 0, 2, 5, 7, 10), cells were trypsinized, resuspended in PBS-EDTA, and counted with a CASY Model TT Cell Counter (Innovatis, Roche Applied Science). Relative cell growth was calculated as the fold change from the baseline day 0 count for each condition.

### Quantitative RT-PCR

RNA was extracted from BMK and MEF cells using the Qiagen RNeasy mini kit or NEB Monarch Total RNA miniprep kit and treated with RNase-free DNase (Qiagen or NEB) prior to cDNA synthesis, both according to the manufacturer's recommendations. cDNA was synthesized using either the high-capacity RNA-to-cDNA kit (Thermo Scientific) or LunaScript RT SuperMix kit (NEB). The qPCR reaction was performed on an ABI 7500 Fast system (Thermo Scientific) or QuantStudio 7 Flex real-time PCR system (Applied Biosystems) using either Fast SYBR GGreen master mix (Thermo Scientific) or PowerUp SYBR Green master mix (Applied Biosystems) according to the manufacturer's recommendations and the primers listed in Supplemental Table S4A. Gene expression was quantified relative to the housekeeping gene *B2m* according to the comparative ΔΔCt method.

For qPCR analysis of p53 targets in mouse tissue samples, liver and duodenum were first isolated and preserved in Allprotect tissue reagent (Qiagen). To make RNA, tissue was homogenized using a Precellys tissue homogenizer (Bertin Instruments) and RNA was extracted using the AllPrep DNA/RNA mini kit (Qiagen), both according to the manufacturers’ recommendations. The resulting DNA was analyzed for recombination as described below. cDNA was synthesized using the high-capacity RNA-to-cDNA kit (Thermo Scientific). The qPCR reaction was performed on a QuantStudio five real-time PCR system (Thermo Scientific) using TaqMan FAST advanced master mix and Taqman gene expression assays (all Thermo Scientific) according to the manufacturer's recommendations and using the assays listed in Supplemental Table S4B. Gene expression was quantified relative to the housekeeping gene *Actin* according to the comparative ΔΔCt method.

### Genotyping PCR

For *Mdm2*^I438K^ MEF and BMK cells and embryos, genomic DNA was isolated as previously described (Wang and Storm 2006). DNA was amplified using KOD hot start PCR master mix (Novagen) according to the manufacturer's recommendations. PCR primers were designed using the Primer BLAST tool (NCBI) and are listed in Supplemental Table S5.

For genotyping of day E12.5 embryos where histology was examined, genomic DNA was isolated using the AllPrep DNA/RNA FFPE kit (Qiagen) according to the manufacturer's recommendations. DNA was then amplified as for MEFs and BMK cells above.

For recombination PCRs, genomic DNA was isolated from mouse liver and duodenum alongside RNA using the AllPrep DNA/RNA mini kit (Qiagen) as described above. DNA was amplified using KAPA2G Fast HotStart genotyping mix according to standard protocols. PCR primers for genotyping and recombination PCRs were designed using the Primer BLAST tool (NCBI) and are listed in Supplemental Table S5.

### Western blotting

Standard procedures were followed for Western blotting. Briefly, proteins were extracted with cell lysis buffer supplemented with EDTA-free Mini-Complete protease inhibitors and a PhosSTOP phosphatase inhibitor cocktail (Roche). Protein lysates were clarified and separated using 4%–12% Bis-Tris NuPAGE gels (Thermo Fisher) and then transferred onto nitrocellulose membranes (GE Healthcare). Membranes were blocked with Odyssey blocking buffer (LI-COR) or 5% dried skimmed milk in TBS-T and incubated with primary antibodies overnight rocking at 4°C. To develop the blots, the membrane was incubated with appropriate IRDye 680LT-conjugated or IRDye 800CW-conjugated secondary antibodies for 1 h before detection using an Odyssey scanner (LI-COR). Images were analyzed using Image Studio software (LI-COR). Alternatively, membranes were incubated with appropriate HRP-linked secondary antibodies (CST) and developed using Pierce ECL Western Blotting Substrate (Thermo Fisher Scientific) and chemiluminescence films (GE Healthcare). ACTIN was used as a loading control performed on the same blot as the other panels shown in each figure. Primary and secondary antibodies used for Western blotting are listed in Supplemental Tables S6A and S7.

### Coimmunoprecipitation

MEFs were transfected with various plasmid pairs (listed in Supplemental Table S3) in a 1:1 ratio using jetPRIME (Polyplus Transfections) transfection reagent according to the manufacturer's instructions. Twenty-four hours after transfection, cells were treated with 10 µM proteasome inhibitor MG-132 (Sigma-Aldrich) for 4 h. Subsequently, protein isolation was carried out using cell lysis buffer (150 mM NaCl, 50 mM Tris-HCl at pH 8.0, 1% Triton X-100) supplemented with protease inhibitor (Roche) (30-min incubation at 4°C followed by two freeze–thaw cycles). From each lysate, an aliquot was saved for input analysis by Western blot while the remaining lysate was incubated with the appropriate antibody (Supplemental Table S6B) on a rotating wheel overnight at 4°C. The next day, 20 µL of Dynabeads Protein G for immunoprecipitation (ThermoFisher Scientific) was added to each lysate followed by a 2-h incubation on a rotating wheel at 4°C. The samples were washed twice with cell lysis buffer before the immunoprecipitates were eluted from the beads with NuPAGE LDS sample buffer (Invitrogen). Samples were boiled for 10 min at 98°C and then loaded on 4%–12% Bis-Tris NuPAGE gels (Thermo Fisher). Western blotting was carried out as described above with appropriate primary antibodies and HRP-linked rabbit anti-mouse IgG (light chain-specific [CSL]) secondary antibody followed by chemiluminescent detection. Antibodies used for immunoprecipitation are listed in Supplemental Table S6B.

### Protein purification

Mouse MDM2 417–C WT and I438K were cloned into pGEX4T1 vector with a N-terminal GST-tag followed by a TEV cleavage sequence and expressed in *Escherichia coli* BL21(DE3) GOLD. Cells were grown in Luria Bertani medium to an OD_600_ of 0.6–1.0 at 37 °C and induced with 0.2 mM isopropyl-β-D-1-thiogalactopyranoside for 16–20 h at 20°C. Cells were lysed in 50 mM Tris-HCl (pH 7.6), 0.4 M NaCl, 1 mM DTT, and 2.5 mM phenylmethylsulfonyl fluoride. GST-MDM2 variants were purified by glutathione Sepharose (GE Healthcare) affinity chromatography followed by gel filtration chromatography. Human UBA1 and fluorescently labeled ubiquitin (Ub) were purified as described previously (Magnussen et al. 2020). The stable UbcH5B-Ub conjugate used in the surface plasmon resonance (SPR) analyses was generated by linking the C terminus of Ub to the catalytic C85S of UbcH5B C85S as described previously (Dou et al. 2012). All protein concentrations were determined by absorbance at 280 nm for Ub and Bio-Rad protein assay with BSA as a standard for other proteins.

### SPR binding analyses

SPR experiments were performed at 25°C on a Biacore T200 instrument using a CM-5 chip (GE Healthcare) with coupled anti-GST antibody as described previously (Magnussen et al. 2020). Briefly, GST-mouse MDM2 variants were coupled on the chip and a serial dilution of UbcH5B-Ub in running buffer containing 25 mM Tris-HCl (pH 7.6), 150 mM NaCl, 0.1 mg/mL BSA, 1 mM DTT, and 0.005% (v/v) Tween-20 was used as analyte. Two technical replicates were performed. The data were analyzed using Biacore T200 BIAevaluation (GE Healthcare) and Scrubber2 (BioLogic Software).

### In vitro autoubiquitination assay

UbcH5B (7 μM) was precharged with UBA1 (0.2 μM) and fluorescently labeled Ub (70 μM) for 30 min at 23°C in buffer containing 50 mM Tris-HCl (pH 7.6), 50 mM NaCl, 5 mM ATP, and 5 mM MgCl_2_. Autoubiquitination reaction was initiated by adding GST-mouse MDM2 417-C variants (0.7 μM) or buffer alone and stopped at the indicated time-point by adding NuPAGE LDS Sample Buffer supplemented with 100 mM DTT. All concentrations in the parentheses indicated final protein concentration in the reaction. The reaction products were separated by SDS-PAGE and ubiquitinated proteins were detected using an Odyssey CLx imaging system (LI-COR Biosciences). The SDS-PAGE was then stained with Coomassie for visualization of E3 loading.

### Data plotting and statistical analysis

Data were plotted using Prism 7 (GraphPad). The statistical analysis for each experiment was performed using the test indicated in the relevant figure legend and multiplicity adjusted *P*-values using the built-in analysis tools of Prism 7. Figures were prepared using Illustrator (Adobe). Unless otherwise indicated, data are represented as mean ± standard error of the mean (SEM) for error bars. Asterisks denote *P*-value as follows: *P* < .05 (*), *P* < .01 (**), *P* < .001 (***), and *P* < .0001 (****).

### Competing interest statement

K.H.V. is on the board of directors and shareholder of Bristol Myers Squibb; a shareholder of GRAIL, Inc. and on the Science Advisory Board (SAB; with stock options) of PMV Pharma, RAZE Therapeutics, and Volastra Therapeutics. She is also on the SAB of Ludwig Cancer. K.H.V. is a cofounder and consultant of Faeth Therapeutics, funded by Khosla Ventures. She has been in receipt of research funding from Astex Pharmaceuticals and AstraZeneca and contributed to CRUK Cancer Research Technology filing of patent application WO/2017/144877. A.K.H. is now an employee of AstraZeneca.

## Supplementary Material

Supplemental Material

## References

[GAD341875HUMC1] Adams DJ, Quail MA, Cox T, van der Weyden L, Gorick BD, Su Q, Chan WI, Davies R, Bonfield JK, Law F, 2005. A genome-wide, end-sequenced 129Sv BAC library resource for targeting vector construction. Genomics 86: 753–758. 10.1016/j.ygeno.2005.08.003 16257172

[GAD341875HUMC2] Andreeff M, Kelly KR, Yee K, Assouline S, Strair R, Popplewell L, Bowen D, Martinelli G, Drummond MW, Vyas P, 2016. Results of the phase I trial of RG7112, a small-molecule MDM2 antagonist in Leukemia. Clin Cancer Res 22: 868–876. 10.1158/1078-0432.CCR-15-0481 26459177 PMC4809642

[GAD341875HUMC3] Booth C, Tudor G, Tudor J, Katz BP, MacVittie TJ. 2012. Acute gastrointestinal syndrome in high-dose irradiated mice. Health Phys 103: 383–399. 10.1097/HP.0b013e318266ee13 23091876 PMC3530834

[GAD341875HUMC4] Chavez-Reyes A, Parant JM, Amelse LL, de Oca Luna RM, Korsmeyer SJ, Lozano G. 2003. Switching mechanisms of cell death in mdm2- and mdm4-null mice by deletion of p53 downstream targets. Cancer Res 63: 8664–8669. 14695178

[GAD341875HUMC5] Cheok CF, Lane DP. 2017. Exploiting the p53 pathway for therapy. Cold Spring Harb Perspect Med 7: a026310. 10.1101/cshperspect.a026310 28193768 PMC5334250

[GAD341875HUMC6] Degenhardt K, Sundararajan R, Lindsten T, Thompson C, White E. 2002. Bax and Bak independently promote cytochrome *C* release from mitochondria. J Biol Chem 277: 14127–14134. 10.1074/jbc.M109939200 11836241

[GAD341875HUMC7] Donehower LA, Harvey M, Slagle BL, McArthur MJ, Montgomery CA Jr, Butel JS, Bradley A. 1992. Mice deficient for p53 are developmentally normal but susceptible to spontaneous tumours. Nature 356: 215-221. 10.1038/356215a0 1552940

[GAD341875HUMC8] Donehower LA, Harvey M, Vogel H, McArthur MJ, Montgomery CA, Park SH, Thompson T, Ford RJ, Bradley A. 1995. Effects of genetic background on tumorigenesis in *p53*-deficient mice. Mol Carc 14: 16–22. 10.1002/mc.2940140105 7546219

[GAD341875HUMC9] Dou H, Buetow L, Sibbet GJ, Cameron K, Huang DT. 2012. BIRC7-E2 ubiquitin conjugate structure reveals the mechanism of ubiquitin transfer by a RING dimer. Nat Struct Mol Biol 19: 876–883. 10.1038/nsmb.2379 22902369 PMC3880866

[GAD341875HUMC10] Fang S, Jensen JP, Ludwig RL, Vousden KH, Weissman AM. 2000. Mdm2 is a RING finger-dependent ubiquitin protein ligase for itself and p53. J Biol Chem 275: 8945–8951. 10.1074/jbc.275.12.8945 10722742

[GAD341875HUMC11] Garcia D, Warr MR, Martins CP, Brown Swigart L, Passegué E, Evan GI. 2011. Validation of mdmX as a therapeutic target for reactivating p53 in tumors. Genes Dev 25: 1746–1757. 10.1101/gad.16722111 21852537 PMC3165938

[GAD341875HUMC12] Grier JD, Yan W, Lozano G. 2002. Conditional allele of mdm2 which encodes a p53 inhibitor. Genesis 32: 145–147. 10.1002/gene.10066 11857803

[GAD341875HUMC13] Habtezion A. 2015. Inflammation in acute and chronic pancreatitis. Curr Opin Gastroenterol 31: 395–399. 10.1097/MOG.0000000000000195 26107390 PMC4618697

[GAD341875HUMC14] Hameyer D, Loonstra A, Eshkind L, Schmitt S, Antunes C, Groen A, Bindels E, Jonkers J, Krimpenfort P, Meuwissen R, 2007. Toxicity of ligand-dependent Cre recombinases and generation of a conditional Cre deleter mouse allowing mosaic recombination in peripheral tissues. Physiol Genomics 31: 32–41. 10.1152/physiolgenomics.00019.2007 17456738

[GAD341875HUMC15] Haupt Y, Maya R, Kazaz A, Oren M. 1997. Mdm2 promotes the rapid degradation of p53. Nature 387: 296–299. 10.1038/387296a0 9153395

[GAD341875HUMC16] Honda R, Yasuda H. 2000. Activity of MDM2, a ubiquitin ligase, toward p53 or itself is dependent on the RING finger domain of the ligase. Oncogene 19: 1473–1476. 10.1038/sj.onc.1203464 10723139

[GAD341875HUMC17] Honda R, Tanaka H, Yasuda H. 1997. Oncoprotein MDM2 is a ubiquitin ligase E3 for tumor suppressor p53. FEBS Lett 420: 25–27. 10.1016/S0014-5793(97)01480-4 9450543

[GAD341875HUMC18] Huang L, Yan Z, Liao X, Li Y, Yang J, Wang ZG, Zuo Y, Kawai H, Shadfan M, Ganapathy S, 2011. The p53 inhibitors MDM2/MDMX complex is required for control of p53 activity in vivo. Proc Natl Acad Sci 108: 12001–12006. 10.1073/pnas.1102309108 21730163 PMC3141917

[GAD341875HUMC19] Humpton TJ, Hock AK, Maddocks ODK, Vousden KH. 2018. p53-mediated adaptation to serine starvation is retained by a common tumour-derived mutant. Cancer Metab 6: 18. 10.1186/s40170-018-0191-6 30524726 PMC6276204

[GAD341875HUMC20] Humpton TJ, Alagesan B, DeNicola GM, Lu D, Yordanov GN, Leonhardt CS, Yao MA, Alagesan P, Zaatari MN, Park Y, 2019. Oncogenic KRAS induces NIX-mediated mitophagy to promote pancreatic cancer. Cancer Discov 9: 1268–1287. 10.1158/2159-8290.CD-18-1409 31263025 PMC6726540

[GAD341875HUMC21] Itahana K, Mao H, Jin A, Itahana Y, Clegg HV, Lindström MS, Bhat KP, Godfrey VL, Evan GI, Zhang Y. 2007. Targeted inactivation of Mdm2 RING finger E3 ubiquitin ligase activity in the mouse reveals mechanistic insights into p53 regulation. Cancer Cell 12: 355–366. 10.1016/j.ccr.2007.09.007 17936560

[GAD341875HUMC22] Iyappan S, Wollscheid HP, Rojas-Fernandez A, Marquardt A, Tang HC, Singh RK, Scheffner M. 2010. Turning the RING domain protein mdmX into an active ubiquitin-protein ligase. J Biol Chem 285: 33065–33072. 10.1074/jbc.M110.115113 20705607 PMC2963378

[GAD341875HUMC23] Jones SN, Roe AE, Donehower LA, Bradley A. 1995. Rescue of embryonic lethality in Mdm2-deficient mice by absence of p53. Nature 378: 206–208. 10.1038/378206a0 7477327

[GAD341875HUMC24] Karni-Schmidt O, Lokshin M, Prives C. 2016. The roles of MDM2 and MDMX in cancer. Annu Rev Pathol 11: 617–644. 10.1146/annurev-pathol-012414-040349 27022975 PMC6028239

[GAD341875HUMC25] Kastenhuber ER, Lowe SW. 2017. Putting p53 in context. Cell 170: 1062–1078. 10.1016/j.cell.2017.08.028 28886379 PMC5743327

[GAD341875HUMC26] Kubbutat MHG, Jones SN, Vousden KH. 1997. Regulation of p53 stability by Mdm2. Nature 387: 299–303. 10.1038/387299a0 9153396

[GAD341875HUMC027] Lee P, Hock AK, Vousden KH, Cheung EC. 2015. p53- and p73-independent activation of TIGAR expression in vivo. Cell Death Dis 6: e1842. 10.1038/cddis.2015.205 26247727 PMC4558498

[GAD341875HUMC27] Levine AJ. 2020. p53: 800 million years of evolution and 40 years of discovery. Nat Rev Cancer 20: 471–480. 10.1038/s41568-020-0262-1 32404993

[GAD341875HUMC28] Linares LK, Hengstermann A, Ciechanover A, Müller S, Scheffner M. 2003. Hdmx stimulates Hdm2-mediated ubiquitination and degradation of p53. Proc Natl Acad Sci 100: 12009–12014. 10.1073/pnas.2030930100 14507994 PMC218704

[GAD341875HUMC29] Liu P, Jenkins NA, Copeland NG. 2003. A highly efficient recombineering-based method for generating conditional knockout mutations. Genome Res 13: 476–484. 10.1101/gr.749203 12618378 PMC430283

[GAD341875HUMC30] Luche H, Weber O, Nageswara Rao T, Blum C, Fehling HJ. 2007. Faithful activation of an extra-bright red fluorescent protein in ‘knock-in’ Cre-reporter mice ideally suited for lineage tracing studies. Eur J Immunol 37: 43–53. 10.1002/eji.200636745 17171761

[GAD341875HUMC31] Ma X, Liu Y, Wang Q, Chen Y, Liu M, Li X, Xiang R, Wei Y, Duan Y, Han J. 2015. Tamoxifen induces the development of hernia in mice by activating MMP-2 and MMP-13 expression. Biochim Biophys Acta 1852: 1038–1048. 10.1016/j.bbadis.2015.02.006 25703139

[GAD341875HUMC32] Magin TM, McWhir J, Melton DW. 1992. A new mouse embryonic stem cell line with good germ line contribution and gene targeting frequency. Nucleic Acids Res 20: 3795–3796. 10.1093/nar/20.14.3795 1641353 PMC334045

[GAD341875HUMC33] Magnussen HM, Ahmed SF, Sibbet GJ, Hristova VA, Nomura K, Hock AK, Archibald LJ, Jamieson AG, Fushman D, Vousden KH, 2020. Structural basis for DNA damage-induced phosphoregulation of MDM2 RING domain. Nat Commun 11: 2094. 10.1038/s41467-020-15783-y 32350255 PMC7190642

[GAD341875HUMC34] Minsky N, Oren M. 2004. The RING domain of Mdm2 mediates histone ubiquitylation and transcriptional repression. Mol Cell 16: 631–639. 10.1016/j.molcel.2004.10.016 15546622

[GAD341875HUMC35] Momand J, Zambetti GP, Olson DC, George DL, Levine AJ. 1992. The mdm-2 oncogene product forms a complex with the p53 protein and inhibits p53-mediated transactivation. Cell 69: 1237–1245. 10.1016/0092-8674(92)90644-R 1535557

[GAD341875HUMC36] Montes de Oca Luna R, Wagner DS, Lozano G. 1995. Rescue of early embryonic lethality in *mdm2*-deficient mice by deletion of *p53*. Nature 378: 203–206. 10.1038/378203a0 7477326

[GAD341875HUMC37] Moyer SM, Larsson CA, Lozano G. 2017. Smdm proteins: critical regulators of embryogenesis and homoeostasis. J Mol Cell Biol 9: 16–25. 10.1093/jmcb/mjx004 PMC5439424 28093454

[GAD341875HUMC38] Nagy A, Gertsenstein M, Vintersten K, Behringer R. 2003. Manipulating the mouse embryo: a laboratory manual, 3rd ed. Cold Spring Harbor Laboratory, Cold Spring Harbor, NY.

[GAD341875HUMC39] Nomura K, Klejnot M, Kowalczyk D, Hock AK, Sibbet GJ, Vousden KH, Huang DT. 2017. Structural analysis of MDM2 RING separates degradation from regulation of p53 transcription activity. Nat Struct Mol Biol 24: 578–587. 10.1038/nsmb.3414 28553961 PMC6205632

[GAD341875HUMC40] Oliner JD, Pietenpol JA, Thiagalingam S, Gyuris J, Kinzler KW, Vogelstein B. 1993. Oncoprotein MDM2 conceals the activation domain of tumour suppressor p53. Nature 362: 857–860. 10.1038/362857a0 8479525

[GAD341875HUMC41] Pant V, Xiong S, Iwakuma T, Quintas-Cardama A, Lozano G. 2011. Heterodimerization of Mdm2 and Mdm4 is critical for regulating p53 activity during embryogenesis but dispensable for p53 and Mdm2 stability. Proc Natl Acad Sci 108: 11995–12000. 10.1073/pnas.1102241108 21730132 PMC3141986

[GAD341875HUMC42] Pant V, Xiong S, Jackson JG, Post SM, Abbas HA, Quintas-Cardama A, Hamir AN, Lozano G. 2013. The p53-Mdm2 feedback loop protects against DNA damage by inhibiting p53 activity but is dispensable for p53 stability, development, and longevity. Genes Dev 27: 1857–1867. 10.1101/gad.227249.113 23973961 PMC3778240

[GAD341875HUMC43] Parant J, Chavez-Reyes A, Little NA, Yan W, Reinke V, Jochemsen AG, Lozano G. 2001. Rescue of embryonic lethality in *Mdm4*-null mice by loss of *Trp53* suggests a nonoverlapping pathway with MDM2 to regulate p53. Nature Genet 29: 92–95. 10.1038/ng714 11528400

[GAD341875HUMC44] Poyurovsky MV, Priest C, Kentsis A, Borden KL, Pan ZQ, Pavletich N, Prives C. 2007. The Mdm2 RING domain C-terminus is required for supramolecular assembly and ubiquitin ligase activity. EMBO J 26: 90–101. 10.1038/sj.emboj.7601465 17170710 PMC1782380

[GAD341875HUMC044] Quast U. 2006. Whole body radiotherapy: a TBI-guideline. J Med Phys 31: 5–12. 10.4103/0971-6203.25664 21206634 PMC3003894

[GAD341875HUMC45] Ringshausen I, O'Shea CC, Finch AJ, Swigart LB, Evan GI. 2006. Mdm2 is critically and continuously required to suppress lethal p53 activity in vivo. Cancer Cell 10: 501–514. 10.1016/j.ccr.2006.10.010 17157790

[GAD341875HUMC46] Rodríguez CI, Buchholz F, Galloway J, Sequerra R, Kasper J, Ayala R, Stewart AF, Dymecki SM. 2000. High-efficiency deleter mice show that FLPe is an alternative to Cre-loxP. Nat Genet 25: 139–140. 10.1038/75973 10835623

[GAD341875HUMC47] Schwenk F, Baron U, Rajewsky K. 1995. A *cre*-transgenic mouse strain for the ubiquitous deletion of *loxP*-flanked gene segments including deletion in germ cells. Nucleic Acids Res 23: 5080–5081. 10.1093/nar/23.24.5080 8559668 PMC307516

[GAD341875HUMC48] Shadfan M, Lopez-Pajares V, Yuan ZM. 2012. MDM2 and MDMX: alone and together in regulation of p53. Transl Cancer Res 1: 88–89. 23002429 PMC3448287

[GAD341875HUMC49] Shi D, Gu W. 2012. Dual roles of MDM2 in the regulation of p53: ubiquitination dependent and ubiquitination independent mechanisms of MDM2 repression of p53 activity. Genes Cancer 3: 240–248. 10.1177/1947601912455199 23150757 PMC3494363

[GAD341875HUMC50] Singh RK, Iyappan S, Scheffner M. 2007. Hetero-oligomerization with mdmX rescues the ubiquitin/Nedd8 ligase activity of RING finger mutants of Mdm2. J Biol Chem 282: 10901–10907. 10.1074/jbc.M610879200 17301054

[GAD341875HUMC51] Tollini LA, Jin A, Park J, Zhang Y. 2014. Regulation of p53 by Mdm2 E3 ligase function is dispensable in embryogenesis and development, but essential in response to DNA damage. Cancer Cell 26: 235–247. 10.1016/j.ccr.2014.06.006 25117711 PMC4369778

[GAD341875HUMC52] Uldrijan S, Pannekoek WJ, Vousden KH. 2007. An essential function of the extreme C-terminus of MDM2 can be provided by MDMX. EMBO J 26: 102–112. 10.1038/sj.emboj.7601469 17159902 PMC1782374

[GAD341875HUMC53] van der Weyden L, Adams DJ, Harris LW, Tannahill D, Arends MJ, Bradley A. 2005. Null and conditional *semaphorin 3B* alleles using a flexible *puroΔtk loxP/FRT* vector. Genesis 41: 171–178. 10.1002/gene.20111 15789413

[GAD341875HUMC54] Vassilev LT, Vu BT, Graves B, Carvajal D, Podlaski F, Filipovic Z, King N, Kammlott U, Lukacs C, Klein C, 2004. In vivo activation of the p53 pathway by small-molecule antagonists of MDM2. Science (New York, NY) 303: 844–848. 10.1126/science.1092472 14704432

[GAD341875HUMC55] Wang X, Jiang X. 2012. Mdm2 and mdmX partner to regulate p53. FEBS Lett 586: 1390–1396. 10.1016/j.febslet.2012.02.049 22673503

[GAD341875HUMC56] Wang Z, Storm DR. 2006. Extraction of DNA from mouse tails. BioTechniques 41: 410–412. 10.2144/000112255 17068955

[GAD341875HUMC57] Wasylishen AR, Lozano G. 2016. Attenuating the p53 pathway in human cancers: many means to the same End. Cold Spring Harb Perspect Med 6: a026211. 10.1101/cshperspect.a026211 27329033 PMC4968169

[GAD341875HUMC58] Watson JV, Chambers SH, Smith PJ. 1987. A pragmatic approach to the analysis of DNA histograms with a definable G1 peak. Cytometry 8: 1–8. 10.1002/cyto.990080101 3803091

[GAD341875HUMC059] Werner H, Karnieli E, Rauscher FJ, LeRoith D. 1996. Wild-type and mutant p53 differentially regulate transcription of the insulin-like growth factor I receptor gene. Proc Natl Acad Sci 93: 8318–8323. 10.1073/pnas.93.16.8318 8710868 PMC38668

[GAD341875HUMC59] Xue J, Sharma V, Hsieh MH, Chawla A, Murali R, Pandol SJ, Habtezion A. 2015. Alternatively activated macrophages promote pancreatic fibrosis in chronic pancreatitis. Nat Commun 6: 7158. 10.1038/ncomms8158 25981357 PMC4632846

[GAD341875HUMC60] Zhang Y, Xiong S, Li Q, Hu S, Tashakori M, Van Pelt C, You MJ, Pageon L, Lozano G. 2014. Tissue-specific and age-dependent effects of global Mdm2 loss. J Pathol 233: 380–391. 10.1002/path.4368 24789767 PMC4151977

[GAD341875HUMC61] Zhou Q, Melton DA. 2018. Pancreas regeneration. Nature 557: 351–358. 10.1038/s41586-018-0088-0 29769672 PMC6168194

