# Supplementary material for: Differential requirements for MDM2 E3 activity during embryogenesis and in adult mice
Source: Genes Dev. 2021 Jan 1;35(1-2):117–32. doi: 10.1101/gad.341875.120 (PMC7778261; doi:10.1101/gad.341875.120)
Supplement: Supplemental Material [file supp_35_1-2_117__DC1.html]

Differential requirements for MDM2 E3 activity during embryogenesis and in adult mice — Supplemental Material 

# Differential requirements for MDM2 E3 activity during embryogenesis and in adult mice

## Supplemental Material

- Supplemental\_Materials\_.pdf
