# Supplementary material for: Differential requirements for MDM2 E3 activity during embryogenesis and in adult mice
Source: Genes Dev. 2021 Jan 1;35(1-2):117–32. doi: 10.1101/gad.341875.120 (PMC7778261; doi:10.1101/gad.341875.120)
Supplement: Supplemental Material [file supp_gad.341875.120_Supplemental_Materials_.pdf]

Supplemental Materials List

Supplemental Figures (S1-S5)

Supplemental Tables

Supplemental Table 1: IHC reagents

Supplemental Table 2: Primary antibodies used in IHC

Supplemental Table 3: Plasmid information

Supplemental Table 4: Quantitative RT-PCR primers

Supplemental Table 5: PCR genotyping primers

Supplemental Table 6: Primary antibodies for western blotting and immunoprecipitation

Supplemental Table 7: Secondary antibodies used in western blotting

Humpton_Supplemental_Fig_1

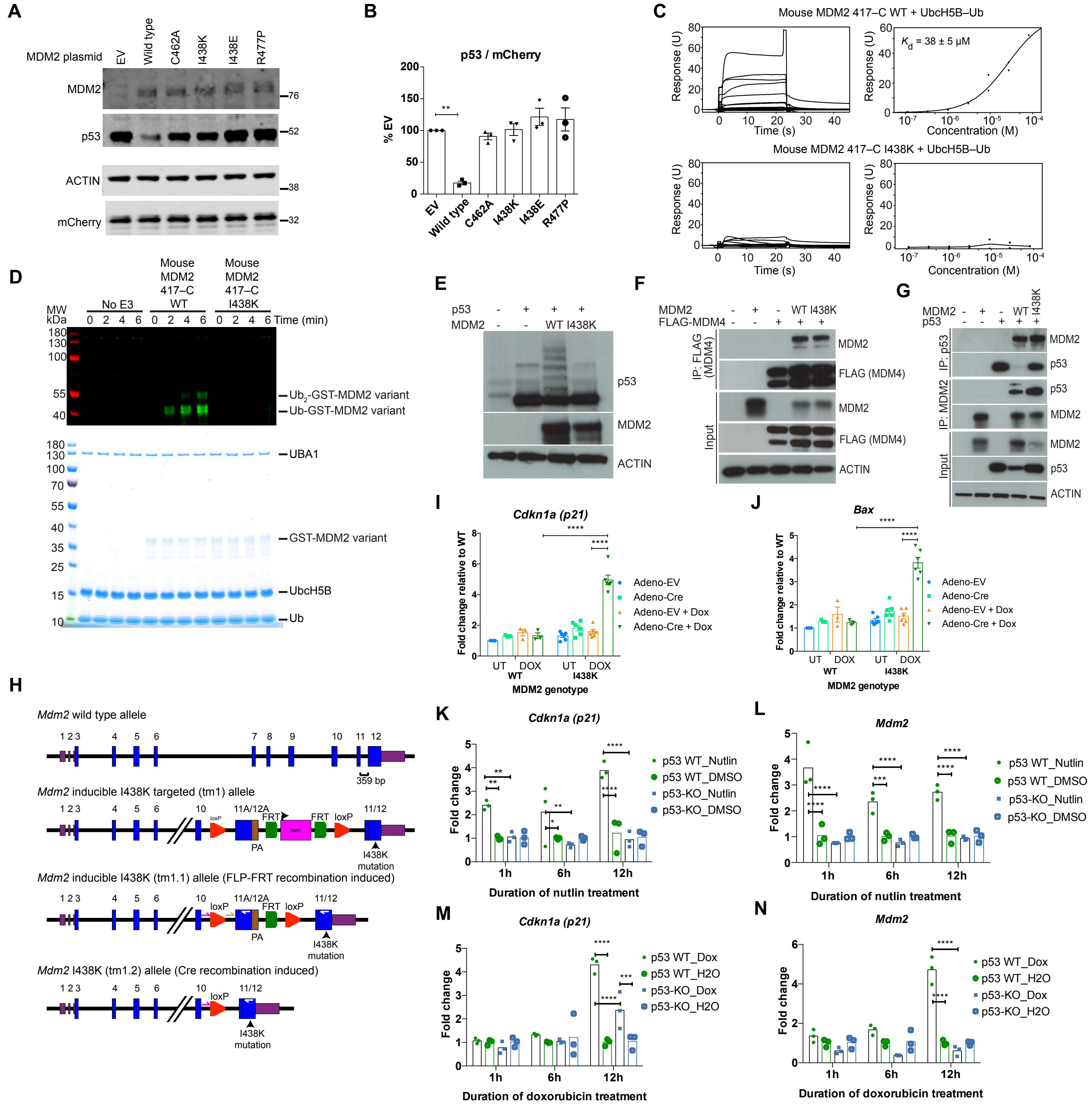

A/B. Western blot analysis (A) and quantification (B) of p53 stability relative to mCherry transfection control in MEFs transfected with the indicated Mdm2 mutants or empty vector control (EV). All proteins detected on one blot with ACTIN used as loading control. Protein MW ladder (kDa) as depicted. One-way ANOVA with Tukey's post-hoc test. Representative blot from 3 independent experiments.

C. SPR analyses of GST-mouse MDM2 variants and UbcH5B–Ub binding affinities. Representative sensorgrams (left) and binding curves (right) for GST-mouse MDM2 variants (indicated) and UbcH5B–Ub are shown. N=2 for each binding curve. Mouse MDM2 417–C WT bound UbcH5B–Ub with a Kd of 38 ± 5 μM, whereas the I438K counterpart had no measurable binding affinity.

D. Reduced SDS-PAGE showing autoubiquitination reaction catalyzed by GST-mouse MDM2 417–C variants using fluorescently-labelled Ub and visualized by an Odyssey CLx Imaging System (top panel) or stained with coomassie (bottom panel). Mouse MDM2 I438K was defective in autoubiquitination.

E. Western blot analysis of p53 to examine ubiquitination in p53KO/KO; Mdm2KO/KO MEFs co-transfected with control plasmids or with p53 and either MDM2 WT or I438K as indicated. Cells were treated with 10μM MG-132 for 5 hours prior to protein isolation. All proteins detected on one blot with ACTIN used as loading control.

F/G. Western blot analysis of co-immunoprecipitation of either MDM2 and MDM4 (F) or MDM2 and p53 (G) in p53KO/KO; Mdm2KO/KO MEFs co-transfected with control plasmids or with MDM4-FLAG (F) or p53 (G) and either MDM2 WT or I438K as indicated. Input and IP samples as indicated. All proteins detected on one blot with ACTIN used as loading control for input. Representative of N=3 independent experiments.

H. Mdm2 I438K knock-in strategy. The inducible Mdm2 I438K targeted allele (tm1.1) contains the Mdm2 WT exons 11/12 (11A/12A) flanked by loxP sites and an FRT-flanked Neomycin selection cassette for ES cell selection. The NEO selection cassette was removed by FLP-FRT recombination (tm1.1 allele). Cre recombination allows for the excision of Mdm2 WT exons 11/12 and the expression of Mdm2 I438K (tm1.2 allele). Arrows indicate location of primers for genotyping and recombination PCR (magenta: recombination forward primer, green: genotyping forward primer, white: universal reverse primer). Further detail in methods.

I/J. RT-qPCR analysis of expression of p53 target genes *Cdkn1a* (*p21*) (I) and *Bax* (J) after 1hr treatment with H2O vehicle control (UT) or 1μM Doxorubicin (DOX) in BMK cells (as in Fig 1B/C). N=3/6 WT/I438K independent samples. Data analysed using 2-way ANOVA with Holm-Sidak's multiple comparisons test and multiplicity-adjusted p-values.

K-N. RT-qPCR analysis of expression of p53 target genes *Cdkn1a* (*p21*) (K/M) and *Mdm2* (L/N) in p53 WT (and Arf-null) and p53 knock-out (KO) MEFs at indicated time points after treatment with either 4μM Nutlin or DMSO control (K/L) or 1μM Doxorubicin (Dox) or H2O vehicle control (M/N). N=3 technical replicates per condition in each experiment shown, representative of N=2 independent experiments. Data analysed using 2-way ANOVA with Holm-Sidak's multiple comparisons test and multiplicity-adjusted p-values.

Humpton_Supplemental_Fig_2

A

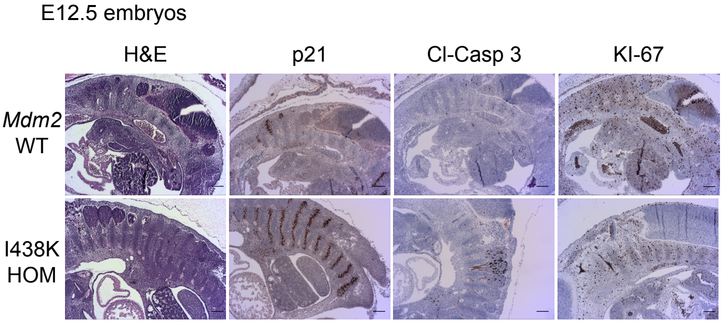

B

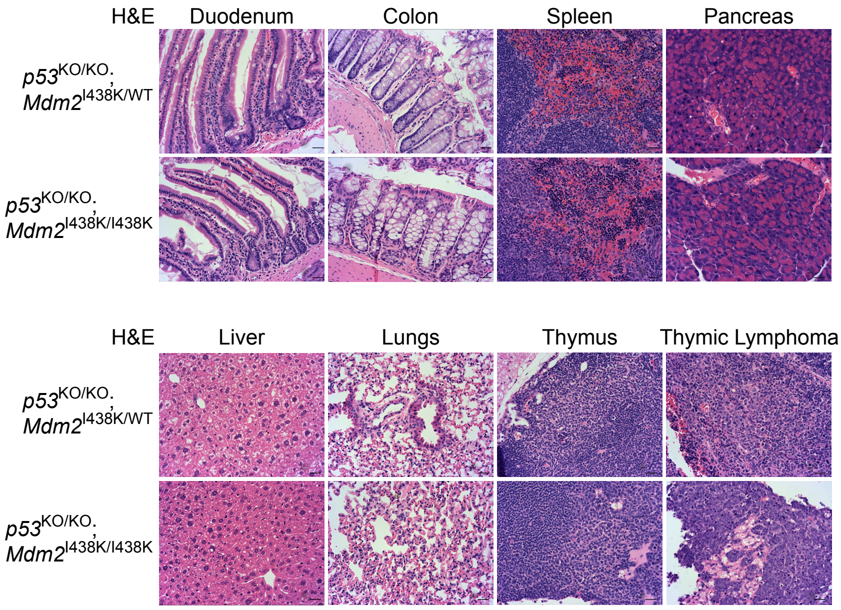

C

***Mdm2*$^{I438K/WT}$; *p53*$^{KO/WT}$ x *Mdm2*$^{I438K/WT}$; *p53*$^{KO/WT}$**

Pups (expected)			
	p53 WT	p53 HET	p53 KO
MDM2 WT	5 (3.3)	7 (6.6)	4 (3.3)
MDM2 I438K HET	13 (6.6)	20 (13.3)	2 (6.6)
MDM2 I438K HOM	0 (3.3)	0 (6.6)	2 (3.3)

Chi-squared	
Chi-sq, df	16.91, 8
P value	0.0311
significance	*

D

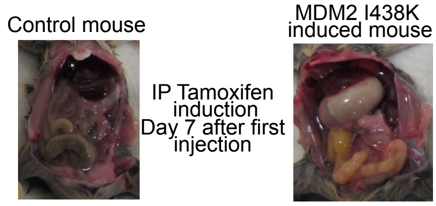

E

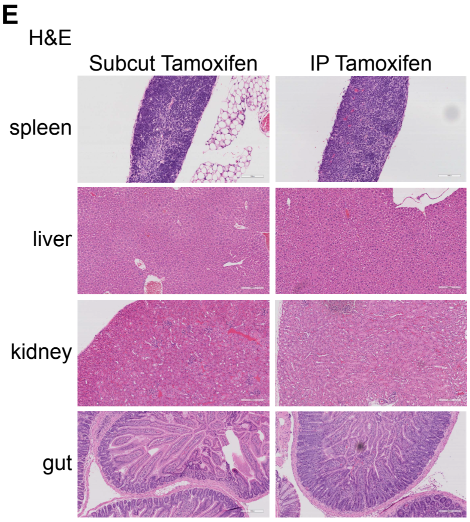

F

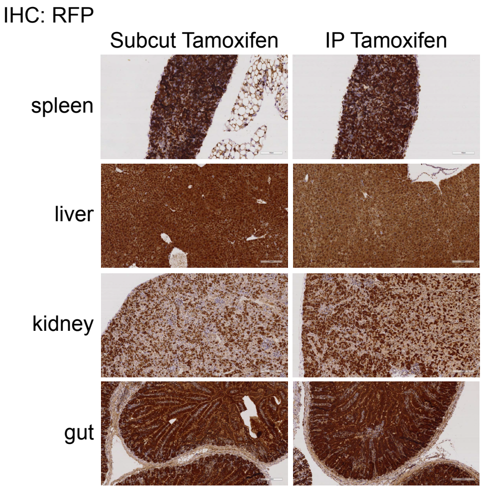

G

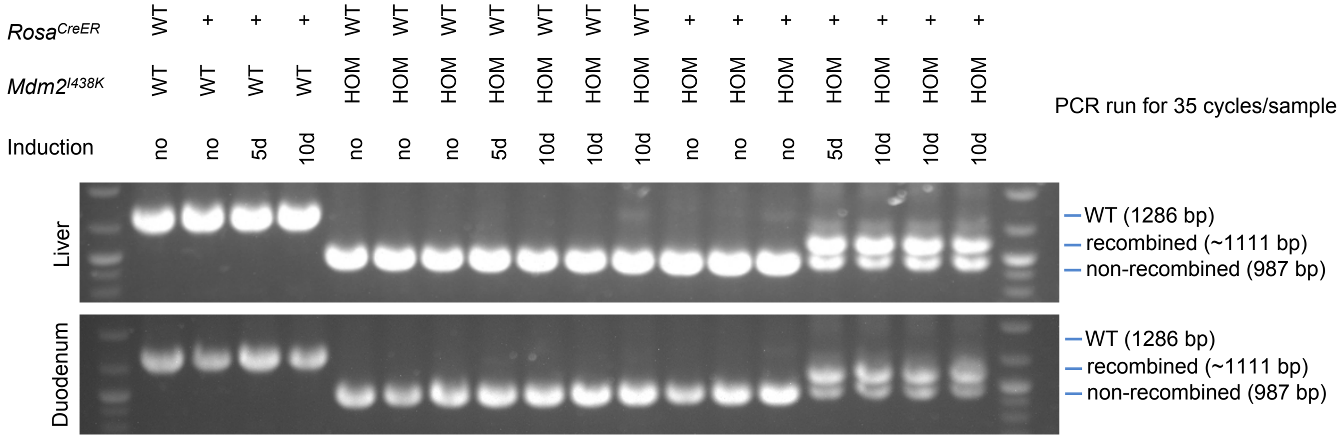

A. Representative H&E and IHC staining for p21, cleaved-Caspase 3 (Cl-Casp 3), and KI-67 in E12.5 *Mdm2* WT/WT (*Mdm2* WT) and *Mdm2* I438K/I438K (I438K HOM) embryos. Representative of N=3 embryos/group derived from three timed matings.
B. Representative H&E staining of indicated tissues comparing *Mdm2* I438K/I438K; *p53* KO/KO and control *Mdm2* I438K/WT; *p53* KO/KO littermate mice at 5 months of age. N=2 mice/genotype. Scale bar 20µm.
C. Genotypes of fifty-three pups born to heterozygous *Mdm2* I438K/WT; *p53* KO/WT matings (offspring from 4 mating pairs) compared with the expected proportions (in parenthesis) according to the Mendelian ratios for a dihybrid cross. Litter data analysed using a Chi-square test to assess lethality of *Mdm2* I438K allele in mice that express p53 as shown.
D. Images from IP cavity of RosaCreER; *Mdm2* I438K/I438K (I438K) and *Rosa*CreER; *Mdm2* WT/WT (WT) mice at 7 days after first IP tamoxifen injection (clinical endpoint for MDM2 I438K mouse). N = 3 mice/genotype.
E. Representative H&E staining of spleen, liver, kidney, and gut from *Rosa*CreER; *R26*-tdRFP Cre reporter mice after either a subcutaneous or IP tamoxifen induction protocol. N=1 mouse/treatment. Scale bar 100µm.
F. Representative IHC staining for RFP in *Rosa*CreER; *R26*-tdRFP Cre reporter mice from (E). Scale bar 100µm.
G. PCR to assess recombination of *Mdm2* I438K allele in the liver and duodenum of *Mdm2* I438K/I438K (HOM) and *Mdm2* WT/WT (WT) mice with or without RosaCreER at 5 and 10 days after SC tamoxifen induction as indicated. Expected bands for WT (1286 bp), recombined (1111 bp), and non-recombined (987 bp) *Mdm2* I438K alleles as shown. PCR reaction run for 35 cycles for all samples.

Humpton_Supplemental_Fig_3

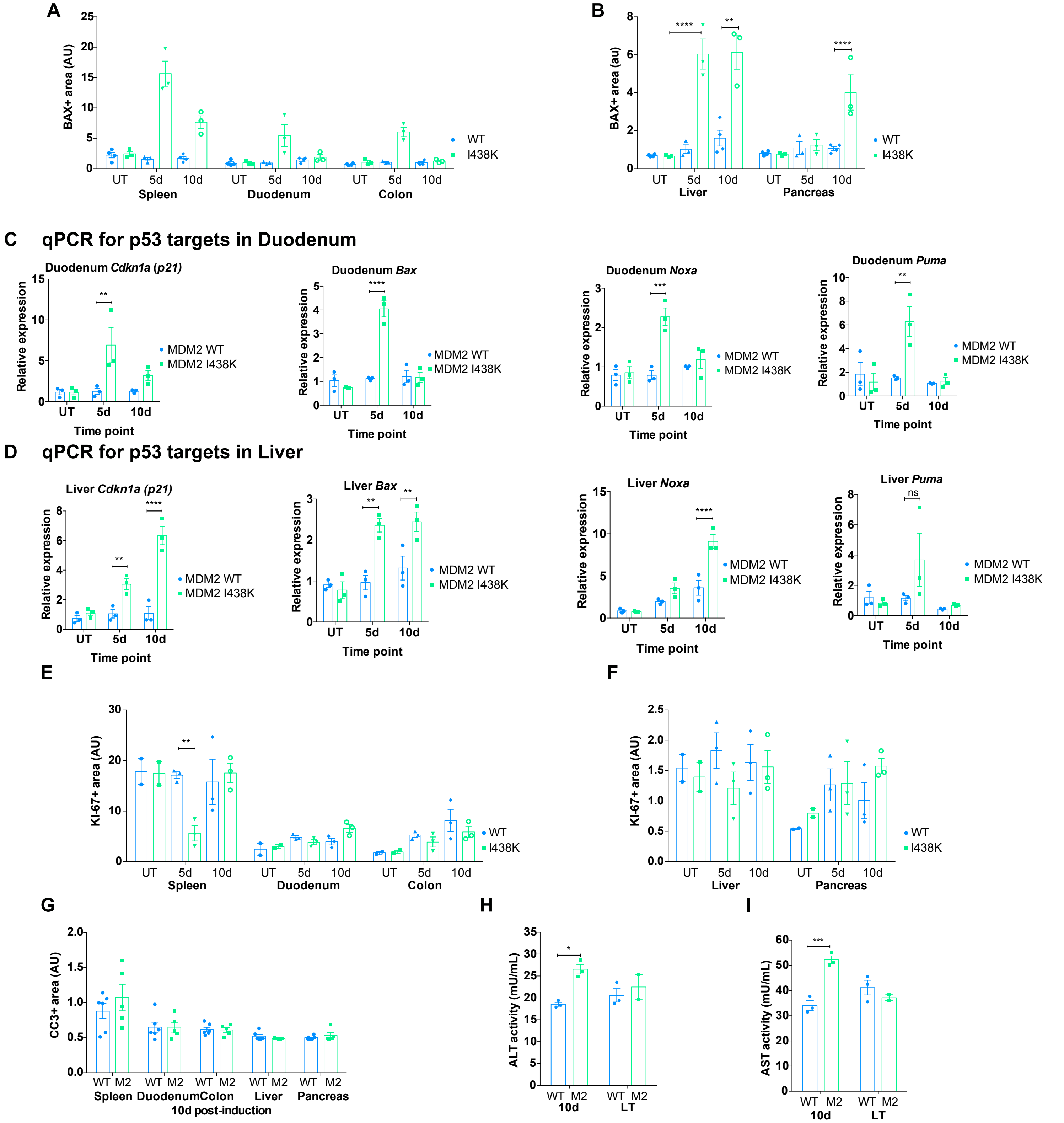

A/B. Quantification of BAX intensity in the indicated proliferative (A) and non-proliferative (B) tissues of *Rosa*CreER; *Mdm2* I438K/I438K (I438K) and *Rosa*CreER; *Mdm2* WT/WT (WT) animals at indicated time points following initial dose of tamoxifen induction (SC). N=4 WT and N=3 I438K uninduced mice, N=3 mice per group at 5 days post-induction and N=4 WT and N=3 I438K mice at 10 days post-induction. Data presented as mean +/- SEM and analysed using 2-way ANOVA with Holm-Sidak's multiple comparisons test and multiplicity-adjusted p-values examining effects of genotype over time after induction within each tissue.
C/D. RT-qPCR analysis of expression of p53 target genes *Cdkn1a (p21)*, *Bax*, *Noxa*, and *Puma* in the duodenum (C) and the liver (D) at indicated time points following initial dose of tamoxifen induction (SC) as in A/B. N=3 mice/group. Data presented as mean +/- SEM and analysed using 2-way ANOVA with Holm-Sidak's multiple comparisons test and multiplicity-adjusted p-values examining effects of genotype over time after induction.
E/F. Quantification of KI-67 intensity in the indicated proliferative (E) and non-proliferative (F) tissues of *Rosa*CreER; *Mdm2* I438K/I438K (I438K) and *Rosa*CreER; *Mdm2* WT/WT (WT) animals as in A/B. N=2 uninduced mice/group and N=3 induced mice/group. Data presented as mean +/- SEM and analysed using 2-way ANOVA with Holm-Sidak's multiple comparisons test and multiplicity-adjusted p-values examining effects of genotype over time after induction in each tissue.
G. Quantification of IHC for cleaved-Caspase 3 in the indicated tissues of *Rosa*CreER; *Mdm2* I438K/I438K (M2) and *Rosa*CreER; *Mdm2* WT/WT (WT) mice sampled at ten days after the first SC tamoxifen injection. N=6 WT and N=5 M2 mice/group. Data presented as mean +/- SEM and analysed using multiple t-tests with Holm-Sidak's method to correct for multiple comparisons.
H/I. Determination of plasma ALT (H), AST (I) (both as mU/mL) in *Rosa*CreER; *Mdm2* I438K/I438K (M2) and *Rosa*CreER; *Mdm2* WT/WT (WT) mice 10 days after the start of tamoxifen induction (10d) and in the longer term (LT). N=3/group except N=2 LT Mdm2 I438K/I438K mice. Mean +/- SEM and data points shown. Data analysed using 2-way ANOVA with Holm-Sidak's multiple comparisons test and multiplicity-adjusted p-values. Data from 10d mice also shown in Figure S5C/D ('no IR', ALT/AST) and from LT mice also shown in Figure 4 M/N (ALT/AST).

A

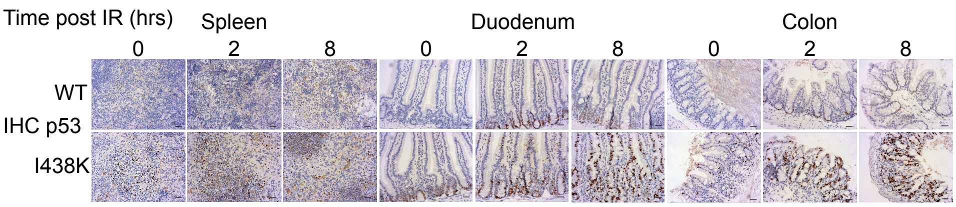

B **qPCR for p53 targets in the Duodenum**

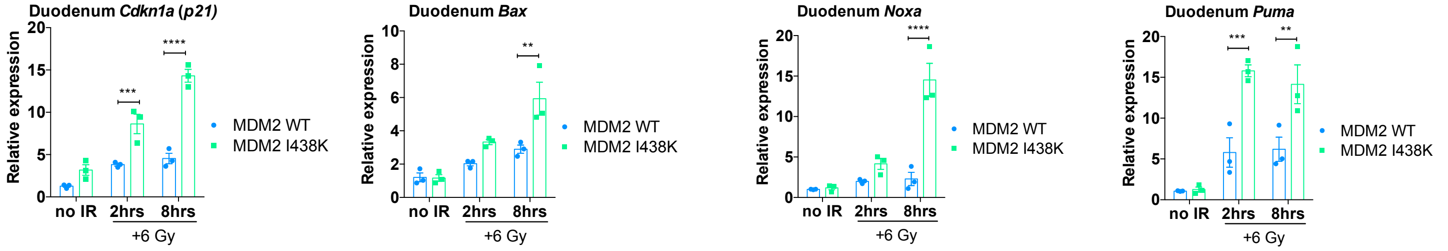

C

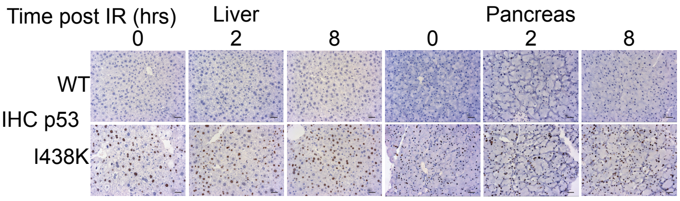

D **qPCR for p53 targets in the Liver**

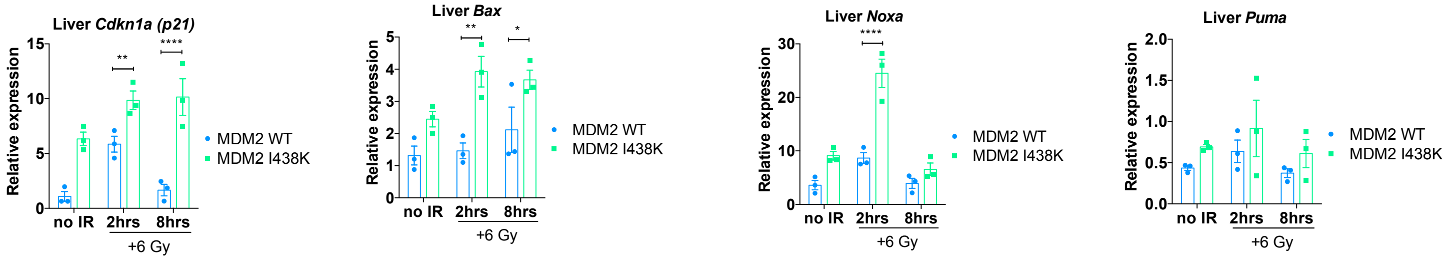

A. Representative IHC staining for p53 of indicated proliferative tissues at the indicated time points (hrs.) after 6 Gy TBI administered 10 days after start of induction in *Rosa*CreER; *Mdm2* I438K/I438K (I438K) and *Rosa*CreER; *Mdm2* WT/WT (WT) mice. Representative of N=6 WT and N=5 I438K control induced mice (no IR) and N=4 WT and N=3 I438K mice per irradiation time point. Scale bar 20μm.

B. RT-qPCR analysis of expression of p53 target genes *Cdkn1a (p21)*, *Bax*, *Noxa*, and *Puma* in the duodenum at indicated time points (hrs.) after 6 Gy TBI as in A. N=3 mice/group. Data presented as mean +/- SEM and analysed using 2-way ANOVA with Holm-Sidak's multiple comparisons test and multiplicity-adjusted p-values examining effects of genotype over time after irradiation.

C. Representative IHC staining for p53 of liver and pancreas as in A.

D. RT-qPCR analysis of expression of p53 target genes *Cdkn1a (p21)*, *Bax*, *Noxa*, and *Puma* in the liver as in B.

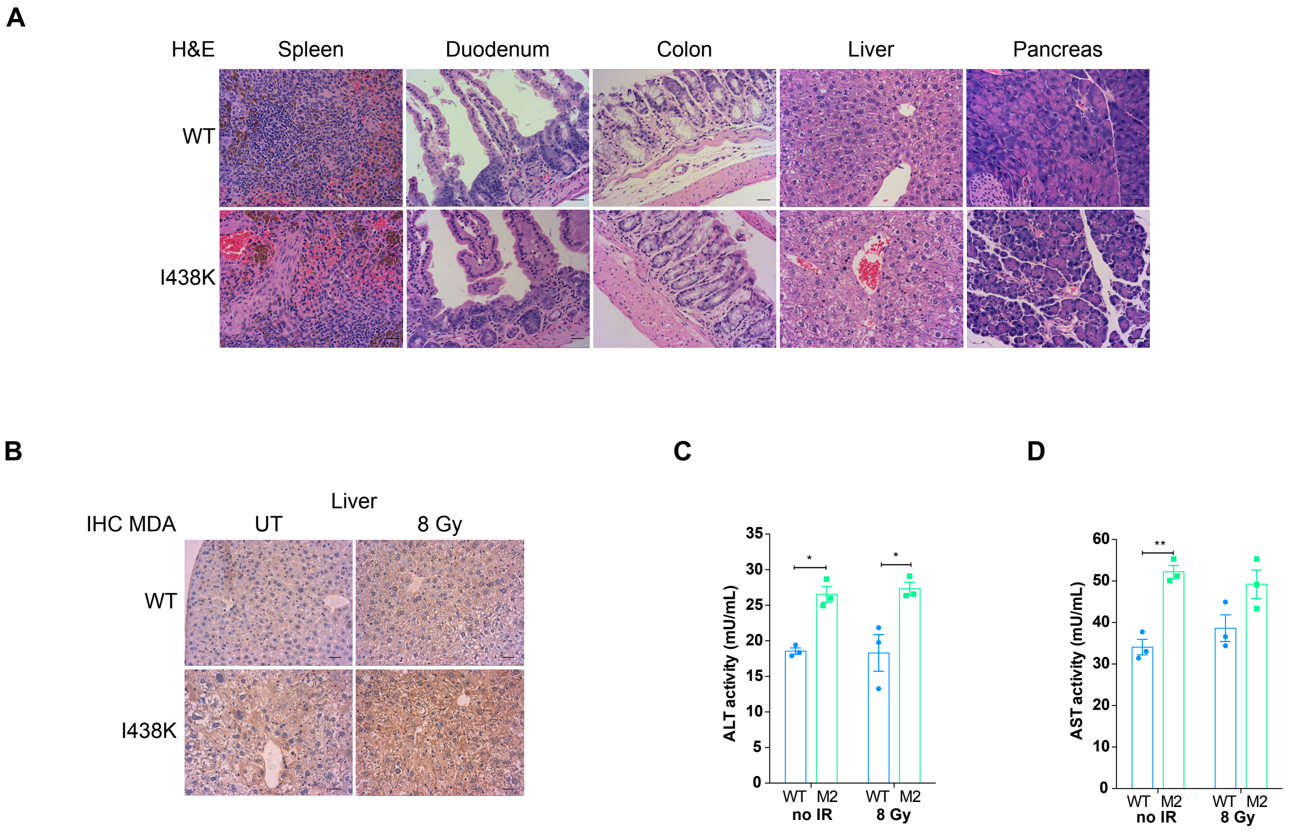

A. Representative H&E images of the indicated tissues 3 days after 8 Gy TBI treatment administered 10 days after start of tamoxifen induction in *Rosa*CreER; *Mdm2* I438K/I438K (I438K) and *Rosa*CreER; *Mdm2* WT/WT (WT) mice. N=4 mice/group. Scale bar 20µm.

B. Representative images for MDA IHC staining in the livers of *Rosa*CreER; *Mdm2* I438K/I438K (I438K) and *Rosa*CreER; *Mdm2* WT/WT (WT) mice that were either analysed 3 days after 8 Gy TBI treatment administered 10 days after start of tamoxifen induction or 10 days after the start of tamoxifen induction alone (UT). N=4 mice/group. Scale bar 20µm (20X).

C/D. Determination of plasma ALT (C) and AST activity (D) (both as mU/mL) in *Rosa*CreER; *Mdm2* I438K/I438K (M2) and *Rosa*CreER; *Mdm2* WT/WT (WT) mice that were either analysed 3 days after 8 Gy TBI treatment administered 10 days after start of tamoxifen induction or 10 days after the start of tamoxifen induction alone (no IR). N=3 mice/group. Data from ‘no IR’ mice also shown in Figure S3 H/I (ALT/AST, ‘10d’). Data analysed using 2-way ANOVA with Holm-Sidak's multiple comparisons test and multiplicity-adjusted p-values.

Supplemental Table 1: IHC reagents

**Reagent**	**Company**	**Code**
Enzyme pre-treatment kit	Leica	AR9551
ER2	Leica	AR9640
Liquid DAB	Agilent	K3468
Rabbit EnVision	Agilent	K4003
Rat ImmPRESS	Vector Labs	MP-7404
TRS High pH antigen retrieval solution	Agilent	K4008
Citrate-based antigen unmasking solution	Vector Labs	H-3300
BLOXALL blocking solution	Vector Labs	SP-6000
Vectastain ABC elite kit	Vector Labs	PK-6010
ImmPACT DAB	Vector Labs	SK-4105
Bond Dewax Solution	Leica	DEWAX-SOLUTION

Supplemental Table 2: Primary antibodies used for IHC

Antibody	Company	Code	Autostainer	Retrieval	Dilution
Caspase 3	CST	9661	Leica Bond Rx	ER2	1:500
F4/80	Abcam	ab6640	Leica Bond Rx	Enz1	1:1000
Phospho-H2A.X	CST	9718	Leica Bond Rx	ER2	1:120
KI67	Abcam	ab16667	Leica Bond Rx	ER2	1:1000
p21	Abcam	ab107099	Leica Bond Rx	ER2	1:150
p53	Leica	NCL-L-p53-CM5p	Dako Link 48	TRS High pH	1:750
RFP	Tebu-Bio	600-401-379	Dako Link 48	TRS High pH	1:1000
MDA	Abcam	ab6463	manual staining	Boiling Citrate	1:333
BAX	CST	5023	Leica Bond RX	ER2	1:2000

Table 3: Plasmid information

Expression	Plasmid	Mutation
EV (empty vector)	pCDNA3.1	n/a
p53 (mouse)	pCDNA3.1	none, WT
MDM2 WT (mouse)	pCDNA3.1	none, WT
MDM2 C462A	pCDNA3.1	MDM2 C462A
MDM2 I438K	pCDNA3.1	MDM2 I438K
MDM2 I438E	pCDNA3.1	MDM2 I438E
MDM2 R477P	pCDNA3.1	MDM2 R477P
FLAG-MDM4 (mouse)	pCMV	none, WT

Table 4A: Quantitative RT-PCR primers used *in vitro*

Gene	Primer	Sequence (5'-3')
B-2 microglobulin	FWD	CGG CCT GTA TGC TAT CCA GA
	REV	GGG TGA ATT CAG TGT GAG CC
Cdkn1a	FWD	CCT GGT GAT GTC CGA CCT G
	REV	CCA TGA GCG CAT CGC AAT C
Mdm2	FWD	CCA ACC ATC GAC TTC CAG CAG CAT T
	REV	GAT TGG CTG TCT GCA CAC TGG G
Bax	FWD	GGA CAG CAA TAT GGA GCT GCA GAG G
	REV	GGA GGA AGT CCA GTG TCC AGC C

Table 4B: Quantitative RT-PCR primers used with tissue samples

Gene	Assay ID
B-Actin	Mm02619580_g1
Cdkn1a/p21	Mm04205640_g1
Bax	Mm00432051_m1
Pmaip1/Noxa	Mm00451763_m1
Bbc3/Puma	Mm00519268_m1

Table 5: PCR genotyping primers

Target	Primer	Sequence (5'-3')	Expected Band Size
Mdm2 I438K	FWD	TTT CCT GCT TGC CTT GAA CTG	1034 bp (WT)
	REV	ATG CAG CCA TTT TTA GGC CG	675 bp (Targeted)
Cre Recombinase	FWD	AGC AAC ATT TGG GCC AGC TA	353 bp
	REV	GGT GCT AAC CAG CGT TTT CG	
I438K Recombination	FWD	CTG GGA TGA AAG GTG TGA GC	1286 bp (WT)<br>987 bp (LSL)<br>1111 bp (recombined)
	REV	ATG CAG CCA TTT TTA GGC CG	

Table 6A: Primary antibodies used for western blotting

Primary Antibody	Company	Code	Dilution
p53 (1C12)	CST	2524	1:1000
MDM2 (Ab-2)	Calbiochem	OP115	1:1000
p21 (C-19)	SCBT	SC-397	1:1000
ACTIN (I-19)	SCBT	SC-1616	1:1000
mCHERRY	Acris Antibodies	AB0040	1:2500
FLAG (M2)	Sigma-Aldrich	F1804	1:1000
ACTIN (13E5)	CST	4970	1:2000

Table 6B: Primary antibodies used for immunoprecipitation

Target	Primary Antibody	Company	Code	Dilution
MDM2	MDM2 (AB-1)	Merck Millipore	OP46	1:100
	MDM2 (Ab-2)	Calbiochem	OP115	1:100
FLAG-MDM4	FLAG (M2)	Sigma-Aldrich	F1804	1:100
P53	P53 (1C12)	CST	2524	1:75

Table 7: Secondary antibodies used for western blotting and immunoprecipitation

**Secondary Antibody**	**Company**	**Dilution**
IRDye 680LT-conjugated	LI-COR	1:15000
IRDye 800CW-conjugated	LI-COR	1:15000
RabbitAnti-Mouse IgG (Light Chain Specific) (HRP conjugate)	CST	1:2500
